# Developing molecular classifiers to detect environmental stressors and smolt stages in sockeye salmon, *Oncorhynchus nerka*

**DOI:** 10.1038/s41598-026-49226-3

**Published:** 2026-06-04

**Authors:** Arash Akbarzadeh, Tobi J. Ming, Shaorong Li, Angela D. Schulze, Oliver P. Günther, Aimee Lee S. Houde, Kristina M. Miller

**Affiliations:** 1 Pacific Biological Station, Fisheries and Oceans Canada, 3190 Hammond Bay Rd, Nanaimo, BC V9T 6N7 Canada; 2 Department of Fisheries, Faculty of Marine Science and Technology, University of Hormozgan, Bandar Abbas, Iran; 3 Günther Analytics, 402-5775 Hampton Place, Vancouver, BC V6T 2G6 Canada; 4 Environmental Dynamics Inc. (EDI), 208A - 2520 Bowen Road, Nanaimo, BC V9T 3L3 Canada

**Keywords:** Salmon, Stressors, Biomarker, Random forest, Fit-chips, Smolt, De-smolt, Climate change, Ecology, Ecology, Environmental sciences, Ocean sciences

## Abstract

Climate change is increasingly affecting Pacific salmon to combinations of thermal, hypoxic, and osmotic stressors that can impair survival. Although genomic tools have enabled detailed insight into stressor responses under controlled conditions, field applications remain limited by the lack of species-specific classifiers capable of resolving physiological condition across multiple stressor contexts. In this study, we developed and experimentally validated transcriptional classifiers for juvenile sockeye salmon (*Oncorhynchus nerka*) using controlled multi-stressor challenges. Fish exposed to a full factorial design of three temperatures (10 °C, 14 °C, 18 °C), three salinities (0, 20, 28 ppt), and two dissolved oxygen levels (> 8 mg L^-1^, 3–3.5 mg L^-1^) over six days, across smolt and de-smolt stages; pre-smolts were additionally challenged with seawater (28 ppt). Gill tissue, a primary interface between fish and their environment, was analyzed using the “Salmon Fit-Chips” microfluidic qPCR tool to quantify gene expression responses. Random Forest models were then used to develop classifiers detecting transcriptional signatures of thermal stress, hypoxic stress, salinity acclimation, and smolt stage. Classifiers achieved high predictive accuracy (85.5%–100%) across all stressors and life stages. Recovery analysis showed that transcriptional stress signals diminished within three days for hypoxia, but lingered longer for thermal stress. By developing sockeye salmon–specific molecular classifiers within a multi-stressor experimental framework, this study advances the Fit-Chip approach and provides a foundation for future field applications, including potential non-lethal monitoring using gill biopsies.

**Supplementary Information:**

The online version contains supplementary material available at 10.1038/s41598-026-49226-3.

## Introduction

Pacific salmon (*Oncorhynchus* spp.) are a crucial focus of scientific research because of their complex anadromous life cycle and their significant ecological, commercial, and cultural importance in North America^1–4^. Unfortunately, a majority of Pacific salmon populations are in decline, with some wild stocks having disappeared entirely^5–7^. This decline has profoundly affected the cultural practices of Indigenous communities, which are deeply intertwined with salmon across generations^8^. Among various human-induced threats, climate change stands out as the primary driver of these population declines^2–4,9,10^. Salmon may experience stress because of high temperature, elevated coastal salinity, and oxygen depletion, but they may also experience stress because of increased infection risks, frequent harmful algal blooms, and the quality and quantity of prey^3,4,8,10–14^.

Sockeye salmon (*Oncorhynchus nerka*) is a Pacific salmon species with a range in North America from the Columbia River to northwestern Alaska^15^. Sockeye salmon is known for its arduous, long distance journeys back to freshwater natal rearing areas that span from 100 to 1,200 km away from the ocean^16^. In British Columbia, sockeye salmon are the most economically valuable commercial salmon species and the second most plentiful among the five species of Pacific salmon in North America^16^. Sockeye salmon consist of both anadromous and non-anadromous ecotypes^17^. The two anadromous ecotypes consist of “lake-type” and “sea-type”^17^. Lake-type sockeye spawn in lakes from late July to early September^11^, with offspring rearing in nursery lakes for 1–2 years before migrating to the ocean, where they grow for another 1–3 years prior to returning to freshwater to spawn^11,17,18^. Sea-type sockeye salmon spawn in tributaries, and the juveniles rear in estuarine waters for several months prior to migrating to the ocean^17^. The third non-anadromous ecotype is commonly known as Kokanee^19^, which carry out their entire life cycle in freshwater^17,19^.

The timing of migration for anadromous juveniles depends on innate factors of the genetic predisposition of the population and body growth and size of an individual, the latter of which can be influenced by stressors encountered in freshwater. Climate change and rising water temperatures, which increase the physiological capacity for body growth in sockeye salmon up to 15 °C^2,18,20^, can influence this timing. Warmer waters lead to faster body growth in juveniles, making them more likely to migrate earlier^11^. However, when water temperatures exceed 15 °C, survival of sockeye salmon decreases rapidly^2^. Moreover, heat stress can impair disease resistance and swim performance, which can reduce individual fitness^21,22^. Climate change-related shifts in water temperature and discharge also have an impact on biotic factors, such as the diversity of macroinvertebrate and planktonic prey species, which in turn has an impact on body growth^12^. Given that higher temperatures are associated with lower dissolved oxygen concentrations, salmon are also increasingly exposed to hypoxic waters^12^. Furthermore, decreased water discharge, an increase in the frequency and intensity of algal blooms, and increasing lake stratification from eutrophication can cause hypoxia in juvenile sockeye salmon^22–24^.

The first few months at sea are when most natural marine mortality occurs in the marine phase of the life cycle of sockeye salmon and other Pacific salmon species^14,26–29^ and can be a key determinate of year-class strength for Pacific salmon species^30^. Challenges faced by sockeye salmon in the ocean that are directly and indirectly related to climate change include: increased acidic and hypoxic waters in nearshore areas; increased abundance of predators; increased incidence of infection and disease; increased frequency and toxicity of harmful algal blooms; decreased thermally suitable habitat area; increased density of competitors for food; decreased zooplankton biomass; and decreased quality of the prey field^12,14,31–36^. These challenging factors can lead to decreased growth of sockeye salmon in the ocean because of higher metabolic demands and higher energy spent on predator avoidance in warm waters^14^. Therefore, the trade-off between the risk of mortality in freshwater environments and size-selective mortality at sea is reflected in the life cycle of sockeye salmon^11^.

In addition to environmental stressors caused by climate change, juvenile survival also depends on timing of smolt migration, and whether it coincides with optimal food availability during early ocean residence^11,12,37,38^. Salmonid smolts must undergo physiological adaptations for seawater entry, such as elevated Na^+^/K^+^-ATPase (NKA) activity, when they enter seawater^38,39^. However, smolts lose their ability to withstand seawater and return to a freshwater form if they are prohibited from accessing seawater during the smolt window^39^. These fish are known as de-smolts^22^. If fish lack the physiological machinery necessary to control ions in a hyperosmotic environment, exposure to seawater beyond the smolt window can be lethal^22,38,39^. Climate change impacts both the timing and duration of the smolt window, with earlier smolt development and a shorter window of optimal seawater readiness with increased temperature^11,37^. Hence, under climate change we predict earlier ocean entry and increased potential that fish migrating long distances to the ocean may revert to de-smolts before they make it to seawater. A more restricted temperature-related smolt window caused by a dam, for example, may further delay migration to the ocean, increasing the frequency of wild de-smolts entering the water and lowering their survival rate^37^.

Early versions of the "Salmon Fit-Chip" technology developed by our group have been successfully applied to investigate stress responses in both freshwater and seawater environments across several salmonid species^24,40,41^. Many of these early applications relied on multivariate approaches such as Principle Component Analysis (PCA) or Non-metric Multidimensional Scaling (NMDS) analyses of biomarker panels combined with known control samples to differentiate stressor profiles. More recently, we initiated a series of multi-stressor challenge studies across multiple salmonid species in the genera *Oncorhynchus*, *Salmo*, and *Salvelinus* to develop species-specific molecular classifiers for key environmental stressors using experimentally generated control fish with known exposure histories^42^. Currently, control fish for thermal and hypoxic stress, salinity acclimation, morbidity status (imminent mortality), and smoltification are available for Chinook salmon^22,43^ and coho salmon^42^. Importantly, these studies employ a consistent experimental framework across species to enable direct comparability of stress responses while ensuring that resulting classifiers are calibrated for each species. For molecular diagnostic tools such as transcriptional biomarker panels and Fit-Chip–based approaches to be applied reliably in ecological monitoring, they must therefore be calibrated and validated using species-specific experimental datasets with known stressor exposure histories. To date, reference datasets for thermal and hypoxic stress, salinity acclimation, morbidity status (imminent mortality), and smoltification have been developed for Chinook salmon^22,43^ and coho salmon^42^. In the present study, we applied a similar experimental design to juvenile sockeye salmon (*O. nerka*). Smolt and de-smolt stages were exposed to combinations of three salinity levels, three temperatures, and hypoxic or normoxic conditions over six days, while pre-smolt fish were exposed to seawater under ambient temperature and dissolved oxygen conditions. Using these multi-stressor challenge trials together with the Salmon Fit-Chip genomic tool and a random forest–based classification approach, we developed classifiers predictive of chronic thermal and hypoxic stress, salinity acclimation, and smolt stage development in sockeye salmon. We evaluated the specificity, sensitivity, and accuracy of different biomarker combinations to diagnostically validate each stressor panel for this species. In addition, we assessed stressor recovery dynamics by applying the classifiers to fish transferred from stressor conditions to ambient environments and sampled daily over three days. By characterizing transcriptional responses of sockeye salmon to controlled environmental stressors, this study aims to improve our ability to detect and interpret physiological responses to multiple stressors in sockeye salmon exposed to complex and variable environmental conditions.

## Materials and methods

### Study species

The multi-stressor challenge study was approved by the Fisheries and Oceans Canada (DFO) Pacific Region Animal Care Committee (PRACC) according to the Canadian Council of Animal Care Standards (AUP#2021–004). All methods were carried out in accordance with guidelines on the care and use of fish in research, teaching and testing (http://www.ccac.ca/Documents/Standards/Guidelines/Fish.pdf), and guidelines of ARRIVE (Home | ARRIVE Guidelines).

Sub-yearling sea-type sockeye salmon (*O. nerka*) fry were transported from Inch Creek Hatchery, Dewdney, British Columbia (BC), Canada to the Pacific Biological Station, Nanaimo, BC, licenced by the BC Introductions and Transfers Committee (ITC) (Licence # 124,710) in May 2021. After transport, sockeye salmon fry were kept in communal circular tanks supplied with dechlorinated ambient municipal freshwater (8 °C -12 °C), aeration, and artificial light set at the natural cycle for almost 1 year until used in the experimental trials. Juveniles were fed pellets at 1–2% of body weight per day (EWOS, Surrey, BC) every day.

### Experimental set-up, welfare monitoring, sampling, and data collection

#### Challenge trials

**Fig. 1:**
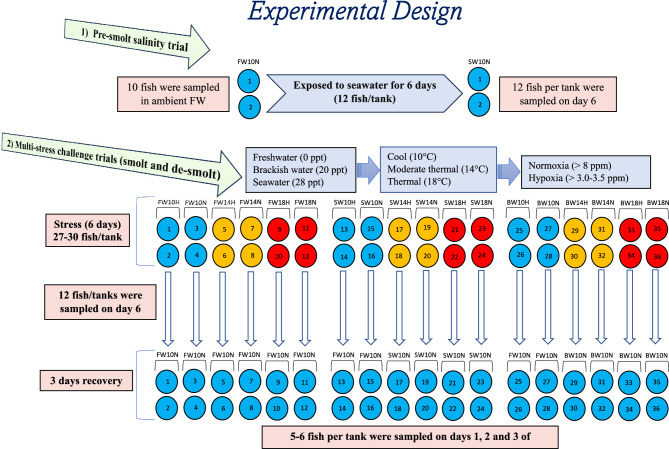
Experimental set-up and tank layout of the 18 groups for three treatments: three salinities by three temperatures by two dissolved oxygen concentrations. FW = Freshwater, BW = Brackish water; SW = Seawater; 10 = 10°C, 14 = 14°C, 18 = 18°C; N = Normoxia, H = Hypoxia.

Three challenge trials were conducted on pre-smolt (March 2022), smolt (May 2022), and de-smolt (June 2022) life stages. In the pre-smolt trial, fish were only exposed to seawater (SW) in ambient temperature and dissolved oxygen (DO) to investigate any osmotic stress signal. The challenge trials on smolt and de-smolt stages were conducted using combinations of three temperatures, i.e., 10 °C (control), 14 °C (moderate thermal stress), and 18 °C (high thermal stress); three salinity values, i.e., 0.0 ppt (freshwater), 20 ppt (brackish), and 28 ppt (seawater); and two dissolved oxygen concentrations, i.e., normoxia (DO > 8.0 mg L^-1^) and hypoxia (DO 3.0–3.5 mg L^-1^, hypoxic stress). Salmon were exposed to 18 treatments with all possible combinations of stressors (Fig. 1).

For each experiment, sockeye salmon were transferred from the communal tanks to the 400 L experimental tanks. For the pre-smolt SW trial, 24 fish were distributed between 2 tanks (12 fish per tank). For the smolt and de-smolt trials, 972 and 1,080 individuals were distributed between 36 tanks, respectively (27 and 30 fish per tank, respectively). In communal tanks, salmon were sedated with 10 ppm Aquacalm and water was treated with Vidalife. Fish were then netted from the communal tanks and lightly anesthetized in a 50 ppm buffered tricaine methanesulfonate (MS-222 or TMS) bath (prepared by dissolving 5 g TMS and 10 g sodium bicarbonate in 100 L of aerated freshwater). After anesthesia, fish were measured for fork length (± 0.1 cm) and mass (± 0.01 g). Condition factor was calculated as K = 100 * W / L3, where W is the fish’s weight in grams (g) and L is its length in centimeters (cm). Surfaces and dip nets in contact with the juveniles were sprayed liberally with concentrated Vidalife. After length and weight measurements, fish were immediately transferred into recovery buckets with aeration and allowed to recover fully before being moved to the experimental tanks. On the transfer day for each trial, gill tissue samples from the right side of 10 fish from the communal tank were placed into SEI buffer (sucrose, EDTA, Imidazol) and then frozen in dry ice for baseline Na^+^/K^+^-ATPase (NKA) activity analysis. NKA activity was measured using the method outlined by McCormick^44^ and conducted at the BC Centre for Aquatic Health Sciences (BC CAHS) in Campbell River, BC. The gill tissue from the left side of these 10 fish was also preserved in RNA later for testing the smolt stage biomarkers. The trial smolt statuses were supported by the traditional use of NKA activity and application of smolt stage biomarkers, many originally developed in Houde et al.^22,43^, but refined herein. Freshwater (12 °C and > 8 mg L^-1^ DO) was supplied to each experimental tank during 7 days of acclimation and fish were fed a 1% body weight ration per day (acclimation conditions).

The challenge trials began with manipulations of the experimental tanks to establish the treatment conditions. For the smaller-scale pre-smolt trial, fish were gradually transitioned over two days from ambient, normoxic freshwater (10 °C) to ambient, normoxic seawater (10 °C; 28 ppt). Following this transition, fish were maintained under seawater conditions for an additional six days and then sampled on day 6. Details of the water manipulations applied during the main multi-stress challenge—designed to induce thermal and hypoxic stress, as well as exposure to saline water (brackish and seawater) in both smolt and de-smolt trials—are provided in Akbarzadeh et al.^42^. In summary, environmental stressor manipulation occurred over two days. On Day 1, brackish water (20 ppt) or seawater (28 ppt) was introduced over 4–5 h in the morning, followed by adjustments to target temperatures of 11 °C, 13 °C, and 16 °C in the early afternoon, while dissolved oxygen (DO) levels were set to 5.0–6.0 mg L⁻1 at 2:00 PM. On Day 2, temperatures were further adjusted to 10 °C, 14 °C, and 18 °C, with DO levels set to 3.0–3.5 mg L⁻1 in the morning. Juveniles were exposed to the full treatment conditions for six days, and their welfare and water quality were monitored every four hours between 6:00 AM and 10:00 PM. No moribund nor dead fish were discovered during the welfare checks.

After six days of full treatment, 12 fish per tank were humanely euthanized using an overdose of TMS (200 ppm), in accordance with PRACC SOP #118. Euthanasia baths were prepared by dissolving 4 g of TMS in 20 L of water. Separate euthanasia baths were prepared for each treatment condition, matching the corresponding temperature, salinity, and dissolved oxygen levels (and buffered for freshwater) to minimize animal stress and discomfort. Fish were immersed in the euthanasia bath until reaching stage IV anesthesia—characterized by complete cessation of opercular (respiratory) movements—typically after approximately 15 min of exposure. Death was confirmed by the absence of opercular movement followed by cardiac arrest before tissue dissection. The euthanized fish were then measured for length and mass, and gill tissue samples were collected from the second gill arch on the left side. All sampled gill tissues were placed in RNAlater, stored initially at 4 °C for 24 h, and then transferred to a -80 °C freezer until analysis for gene expression.

#### Recovery trials

After Day 6, the remaining fish from each treatment were gradually returned to ambient conditions (normoxic and 10 °C) over a period of 4–5 h to allow recovery from the environmental stressors for three days. The recovery conditions for each treatment are illustrated in Fig. 1. To assess the fish’s ability to re-acclimate to freshwater, those acclimated to seawater (SW, 10 °C) and brackish water (BW, 10 °C) were transitioned back to freshwater (FW, 10 °C) over 4–5 h. Following this transition, six fish per tank were sampled each day after recovery periods of 12 h (Day 1), 36 h (Day 2), and 60 h (Day 3), totaling 18 fish per tank. Sampling methods were consistent with those used in the challenge trials.

### Gene expression

A total of 1,240 individuals were examined for gene expression analysis of gill tissue. The dataset included 35 fish sampled at the pre-smolt stage (11 in freshwater and 24 in seawater), 689 fish from the smolt trial (251 at day 6 of full treatment and 438 sampled during the three recovery days), and 516 fish from the de-smolt trial (196 at day 6 of full treatment and 320 sampled during the three recovery days). As no mortality was observed within any of the three trials, validation of the morbidity biomarker panel was not pursued.

Gill tissues were homogenized using TRIzol (Ambion, Foster City, CA, USA) and BCP reagent (Sigma-Aldrich, Oakville, ON, Canada). RNA was extracted with the MagMAX-96 Total RNA Isolation kits (Ambion), normalized to 62.5 ng/mL, and cDNA was synthesized using SuperScript VILO synthesis kits (Invitrogen). For more details, refer to Akbarzadeh et al.^45^, Houde et al.^22^, Akbarzadeh et al.^45^, Akbarzadeh et al.^24,25^, and Akbarzadeh et al.^42^.

Gene expression was analyzed for 94 curated biomarkers related to thermal and hypoxic stress, osmoregulation, smoltification, morbidity status, and viral disease development^22,42,45–47^, along with two assays for screening the Saprolegnia parasite (Supplementary Table 1). As described in Akbarzadeh et al.^42^, target cDNA amplicons for each gene were first enriched with the specific target amplification (STA) method, and then all the amplified samples and the 96 TaqMan assays (primers and probes) were run on 96.96 gene expression dynamic arrays (chips) on the microfluidics Fluidigm BioMark HT platform (Fluidigm Corporation, CA, USA). A total of 16 dynamic arrays were utilized, each also contained serial dilutions (1, 1/5, 1/25, 1/125, 1/625, and 1/3,125) of a cDNA pool comprised of all sockeye salmon samples. Detailed information regarding sample and assay reactions loaded onto the microfluidics dynamic arrays and Real-Time PCR conditions can be found in Akbarzadeh et al.^42^. Data extraction was performed using the Real-Time PCR Analysis Software (Fluidigm), and the Ct thresholds were manually scored for each assay across all chips.

PCR efficiency for each assay per chip was calculated using (10 ^1/slope^—1) × 100, where the slope was derived from a plot of Ct values against the serial dilutions of cDNA. To identify the optimal normalization genes, the expression levels of all 94 biomarkers were assessed across the 16 chips using the software tools of geNorm, NormFinder, and BestKeeper (see Akbarzadeh et al.^42^). The expression levels of the samples for each chip run were then normalized to the geometric mean of HP_GP_609 (Glycogen phosphorylase), VDD_NFX_87 (Zinc finger NFX1-type), and VDD_TRIM1_409 (Fish virus induced TRIM-1) — identified as the most suitable normalization genes. Normalization was performed using the ∆∆Ct method^48^, employing the pooled sample from each chip as the calibrator to account for chip-to-chip variation in GenEx (ver. 7). Finally, gene expression values were log transformed: using the formula log_2_ (2^-∆∆Ct^) for all further analyses.

### Data exploratory and classification analyses

The exploratory and classification analyses of the data are detailed in Akbarzadeh et al.^42^. Briefly, assay efficiencies for the genes ranged from 0.8 to 1.2, which were deemed suitable for further analyses (see Supplementary Table 2). Then the entire normalized gene expression dataset was split randomly into analytical training (two-thirds samples) and testing datasets (one-third samples). The training dataset was first subjected to exploratory analyses, including T-tests (with the Mann–Whitney non-parametric test applied for biomarkers that did not meet the normality assumption), Receiver Operating Characteristic (ROC) analyses, and ANOVA/Tukey’s HSD tests (FDR-adjusted p-values used to consider significant biomarkers for multiple comparisons). These analyses aimed to identify which biomarkers contributed most to the statistically significant differentiation of each stress response or physiological signal prior to the classification analysis. Next, the initial RF-based classification model was applied to the training dataset using the statistically significant biomarkers, and GINI scores were used to rank each biomarker for contribution to the differentiation of stressor or physiological states. The model was then tested on the testing dataset to calculate performance parameters, including sensitivity, specificity, and prediction accuracy. Several iterations of RF classifier development were conducted by removing biomarkers with the lowest Gini scores until the sensitivity and accuracy performance on testing dataset started to decline. The final RF analysis for each classifier used the best-performing combination of biomarkers, as determined by the highest performance metrics from the initial RF analysis.

To evaluate recovery times from thermal and hypoxic stress, as well as from seawater acclimation, the previously trained optimised RF-based model was applied to fish during the recovery trial. This included fish returning from 18 °C and 14 °C to 10 °C, transitioning from hypoxia to normoxia, and acclimating from 10 °C seawater (SW) back to 10 °C freshwater (FW).

The RF-based classifiers were developed using the Random Forest R package, based on the algorithm by Breiman and Cutler^49^. We applied PCA to each biomarker panel used in the final RF analysis to visualize group separation, utilizing the fviz_pca function from the factoextra R package. This function generated 95% confidence ellipses for the groups.

## Results

The sample size, mean body length, mass, and condition, as well as Na⁺/K⁺-ATPase activity for pre-smolt, smolt, and de-smolt fish are presented in Table 1. The mean body weights were 38.5 g, 57.8 g, and 72.9 g for pre-smolt, smolt, and de-smolt stages, respectively. The condition factor ranged from 1.13 to 1.19 across the different smolt stages. Mean body length and mass increased in line with the life stage for the trials, while the body condition was similar across the trials. As expected, Na⁺/K⁺-ATPase activity increased significantly from the pre-smolt to smolt stage. A decreasing trend was also observed in the de-smolt stage compared to smolt fish; however, this difference was not statistically significant (see Table 1). No mortality was observed in any trial during the exposure or recovery periods.

**Table 1:** Summary of the trial date, mean fish body length, mass and condition factor ± S.D, smolt status, and gill Na^+^/K^+^-ATPase activity (μmol ADP (mg protein)^-1^ h^-1^).

Trial	Date	N	Length (cm)	Mass (g)	Condition factor	Na^+^/K^+^-ATPase
Pre-smolt	16–29 March, 2022	34	15.3 ± 0.5	38.5 ± 4.4	1.13 ± 0.11	4.3 ± 1.5 b
Smolt	03–22 May, 2022	972	16.9 ± 0.9	57.8 ± 9.4	1.19 ± 0.08	9.8 ± 2.8 a
De-smolt	07–26 June, 2022	1080	18.4 ± 0.9	72.9 ± 17.3	1.17 ± 0.23	7.4 ± 0.42 a

Na^+^/K^+^-ATPase data (mean ± SD) with different letters are significantly different among different smolt stages (*p* < 0.05; ANOVA/ Tukey’s HSD tests).

### Thermal stress classifier

**Fig. 2:**
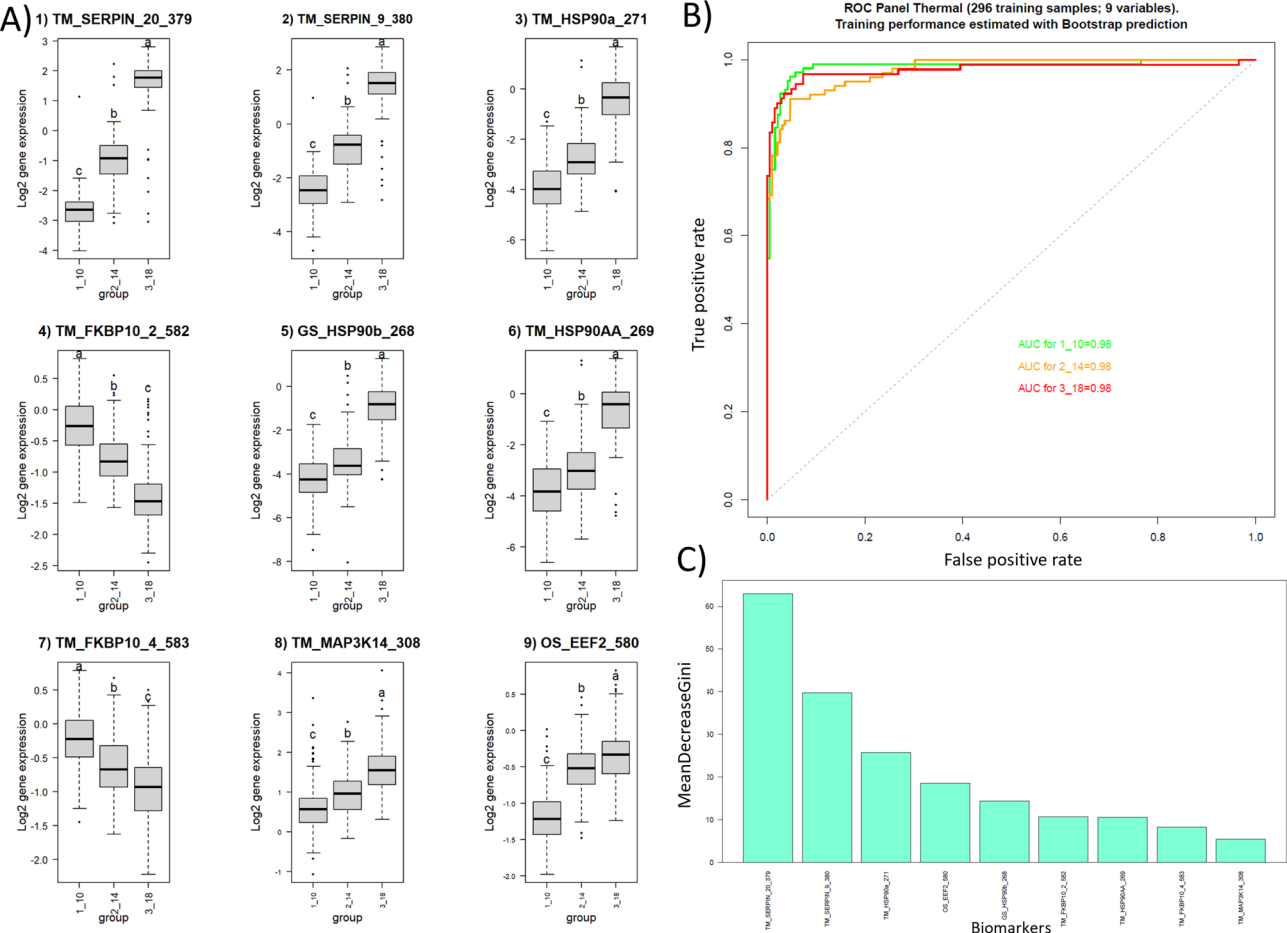
(**A**) The mRNA expression patterns of 9 selected thermal stress biomarkers for RF analysis among 3 temperatures of 10°C, 14°C and 18°C. (**B**) Receiver Operating Characteristic ROC curve analysis of the RF model in the train dataset for thermal stress classifier. There are three curves showing results for each of the three temperature categories (positive class) vs. the rest (negative class), so the figure shows results for three classifiers, while the 3-category thermal classifier score would include the results for a single (3-class) classifier. The 3-class classifier might select a temperature category even though the score is only 0.4 (for example if the other two categories each have a score of 0.3). (**C**) The biomarker rank for thermal stress classifier based on mean decrease in accuracy and Gini scores.

Nine biomarkers were required to optimize specificity and sensitivity for detecting thermal stress during the challenge trials (supplementary Table 3), classifying with high accuracy the fish in the 10 °C, 14 °C, and 18 °C treatments. The mRNA expression patterns for each biomarker among 3 temperatures are shown in Fig. 2A. Seven biomarkers were significantly upregulated from 10 °C to 14 °C and 14 °C to 18 °C (*p* < 0.05; ANOVA/ Tukey’s HSD tests), while two biomarkers were significantly downregulated from 10 °C to 14 °C and 14 °C to 18 °C (*p* < 0.05; ANOVA/ Tukey’s HSD tests).

The RF-based model for the thermal stress classifier on the training dataset (N = 296) showed an area under the ROC curve (AUC) of 98% for the 10 °C, 14 °C, and 18 °C treatments, (Fig. 2B). On the testing dataset (N = 145), the model showed high prediction accuracy of 97.9%, 96.6% and 98.6% for the 10 °C, 14 °C, and 18 °C treatments, respectively (Table 2). The sensitivity scores were 100%, 90%, and 100% for the 10 °C, 14 °C, and 18 °C treatments, respectively (Table 2). TM_SERPIN20_379, TM_SERPIN9_380, and TM_HSP90a_271 were the most important thermal stress biomarkers in the model based on the Gini scores (Fig. 2C), with powerful up-regulation in 18 °C fish (Fig. 2A).

**Table 2:** Performance analysis of the RF model on the one-third testing dataset (day 6 stressed samples) for thermal stress, hypoxic stress, salinity acclimation, and smolt stage classifiers using threshold of 0.5.

Classifiers	Treatments	Threshold 0.5		
		sensitivity %	Specificity %	Prediction accuracy %
Thermal stress	1–10 °C 2–14 °C 3–18 °C	100.0 90.0 100.0	96.7 100.0 98.1	97.9 96.6 98.6
Hypoxic stress	1_Normoxia 2_Hypoxia	87.7 83.3	83.3 87.7	85.5
Salinity acclimation	1_Freshwater 2_Seawater	100.0 100.0	100.0 100.0	100.0
Smolt stage	1-Pre-smolt 2-Smolt 3- De-smolt	72.7 88.1 88.5	98.6 87.5 90.5	96.5 87.7 89.5

The RF-based modeling applied during the recovery trials indicated that nearly 90% of fish from the 14 °C treatment were classified as belonging to the 10 °C treatment after 3 days (60 h) of being returned to 10 °C (Table 3). In contrast, after 3 days of recovery from 18 °C to 10 °C, only approximately 10% of fish were classified as the 10 °C treatment, while almost 90% were classified as the 14 °C treatment.

**Table 3:** The predicted class frequencies during three recovery days from thermal stress, hypoxic stress and seawater recovery when fish were introduced back to 10°C from 14°C and 18°C, from hypoxia (H) to normoxia (N), and from 10°C seawater(SW) to 10°C freshwater (FW), respectively.

Thermal stress recovery	N	10 °C	14 °C	18 °C	Hypoxic stress recovery	N	Normoxia	Hypoxia	Seawater recovery	N	FW	SW
14 to 10°C day1	84	14	63	7	H to N day1	136	81	55	SW to FW day1	24	3	23
14 to 10°C day2	92	54	37	1	H to N day2	134	75	59	SW to FW day2	27	14	13
14 to 10°C day3	78	69	8	1	H to N day3	124	61	63	SW to FW day3	24	23	1
18 to 10°C day1	81	4	20	57								
18 to 10°C day2	94	4	86	4								
18 to 10°C day3	80	9	70	1								
	**%**					**%**				**%**		
14 to 10°C day1	100	16.7	75.0	8.3	H to N day1	100	59.6	40.4	SW to FW day1	100	12.5	87.5
14 to 10°C day2	100	58.7	40.2	1.1	H to N day2	100	56.0	44.0	SW to FW day2	100	51.9	48.2
14 to 10°C day3	100	88.5	10.3	1.2	H to N day3	100	49.2	50.8	SW to FW day3	100	95.8	4.2
18 to 10°C day1	100	4.9	24.7	70.4								
18 to 10°C day2	100	4.3	91.5	4.2								
18 to 10°C day3	100	11.3	87.5	1.2								

**Fig. 3:**
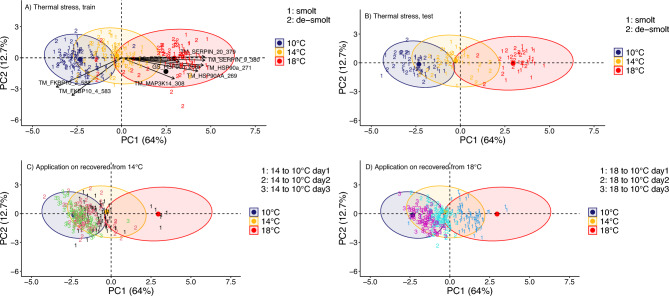
Canonical plots of the first two principal components of the 9 optimal biomarkers for thermal stress. Principal component analysis was performed on the 2/3 training set (**A**) and then tested to the 1/3 testing set (**B**). PCA was also applied on the 3 days recovery from 14 to 10°C set (**C**), and on the 3 days recovery from 18 to 10°C set (**D**). Ellipses represent 95% confidence areas for the groups within treatments using the training set; centroids are represented by the largest point of the same colour. Arrows (left panel) represent loading vectors of the biomarkers using the training set. Data with different letters are significantly different among different smolt stages (*p* < 0.05; ANOVA/ Tukey’s HSD tests).

The PCA based on the 9 optimal genes contributing to the thermal stress classification displayed visible separation of the 10 °C and 18 °C groups. Fish from the lowest (10 °C) and highest temperatures (18 °C) separated with some overlap from 14 °C. The PCA plot also displays that following 3 days of recovery from 14 °C to 10 °C, almost all fish cluster within the 10 °C ellipse (Fig. 3). On the other hand, from 18 °C to 10 °C, almost all fish cluster within the overlap of 10 °C and 14 °C ellipses.

### Hypoxic stress classifier

**Fig. 4:**
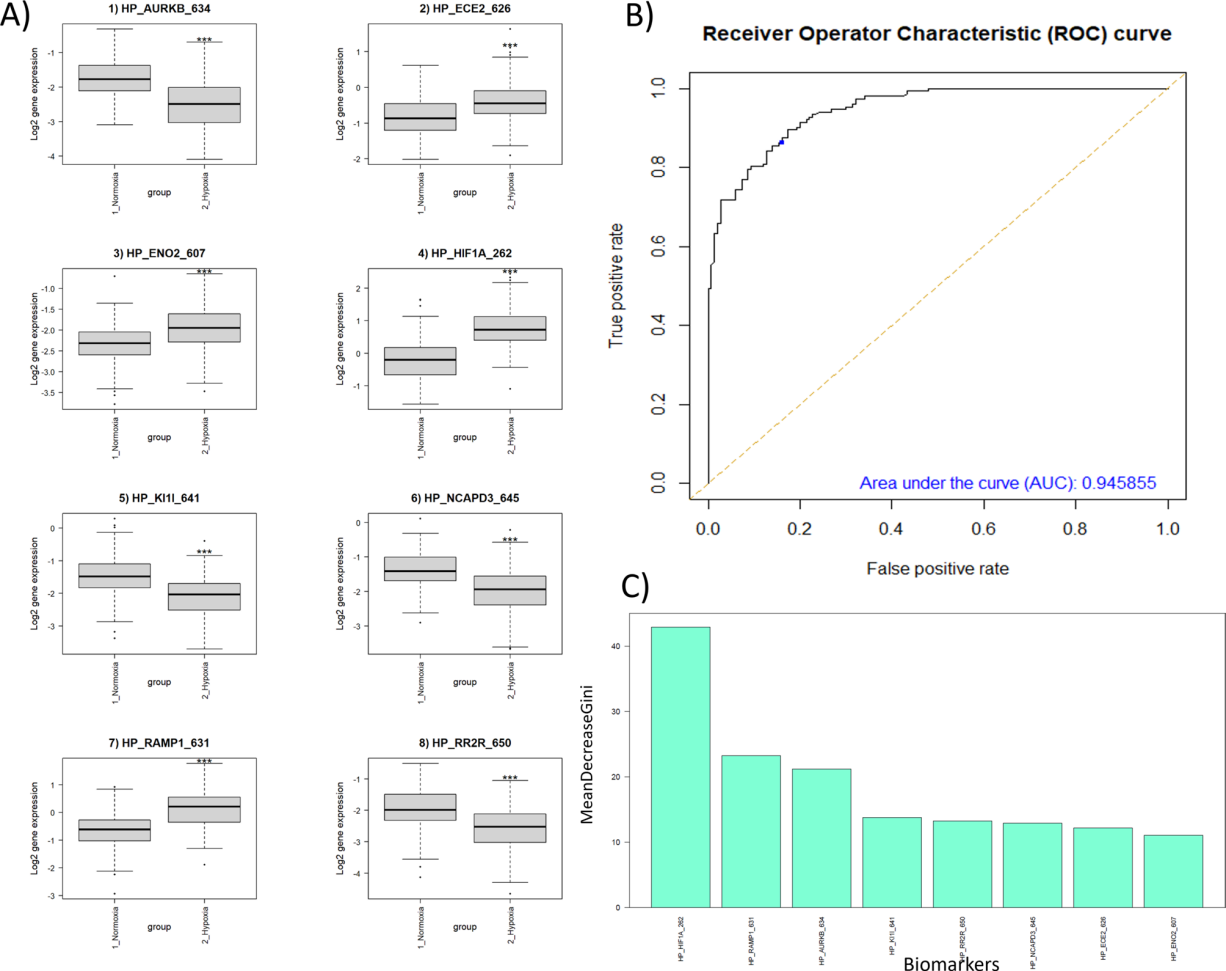
(**A**) The mRNA expression patterns of 8 selected hypoxic stress biomarkers for RF analysis between hypoxia and normoxia groups (**B**) Receiver Operating Characteristic ROC curve analysis of the RF model in the train dataset for hypoxia stress classifier. (**C**) The biomarker rank for hypoxic stress classifier based on mean decrease in accuracy and Gini scores. t-test: *** p < 0.001.

A panel of eight hypoxic stress biomarkers effectively classified fish into hypoxia and normoxia treatments during the challenge trials (Supplementary Table 4). The mRNA expression patterns for each biomarker between the two treatments are displayed in Fig. 4A. In hypoxia, four biomarkers were significantly upregulated and four were downregulated compared to normoxia (*p* < 0.001; t-test).

The RF-based model for the hypoxic stress classifier demonstrated an area under the ROC curve (AUC) of 95% on the training dataset (N = 302) (Fig. 4B). In the testing dataset (N = 145), the model achieved a prediction accuracy of 85.5% for hypoxia and normoxia treatments, with sensitivity scores of 87.7% for normoxia and 83.3% for hypoxia (Table 2). The most important hypoxic stress biomarkers in the model, as indicated by Gini scores, were HP_HIF1A_262, HP_RAMP1_631, and HP_AURKB_634 (Fig. 4C).

The RF-based modeling applied to fish during the recovery trials revealed that nearly 40% of fish previously exposed to hypoxia in the challenge trials were classified as normoxia after just one day of returning to normoxia (recovery trials; Table 3). However, after three days of recovery, nearly half of the fish returned to normoxic water still displayed a hypoxia signal (Table 3).

The PCA displays separation of the normoxia and hypoxia treatments with some overlap of the ellipses between the two groups (Supplementary Fig. 1).

### Salinity acclimation classifiers

**Fig. 5:**
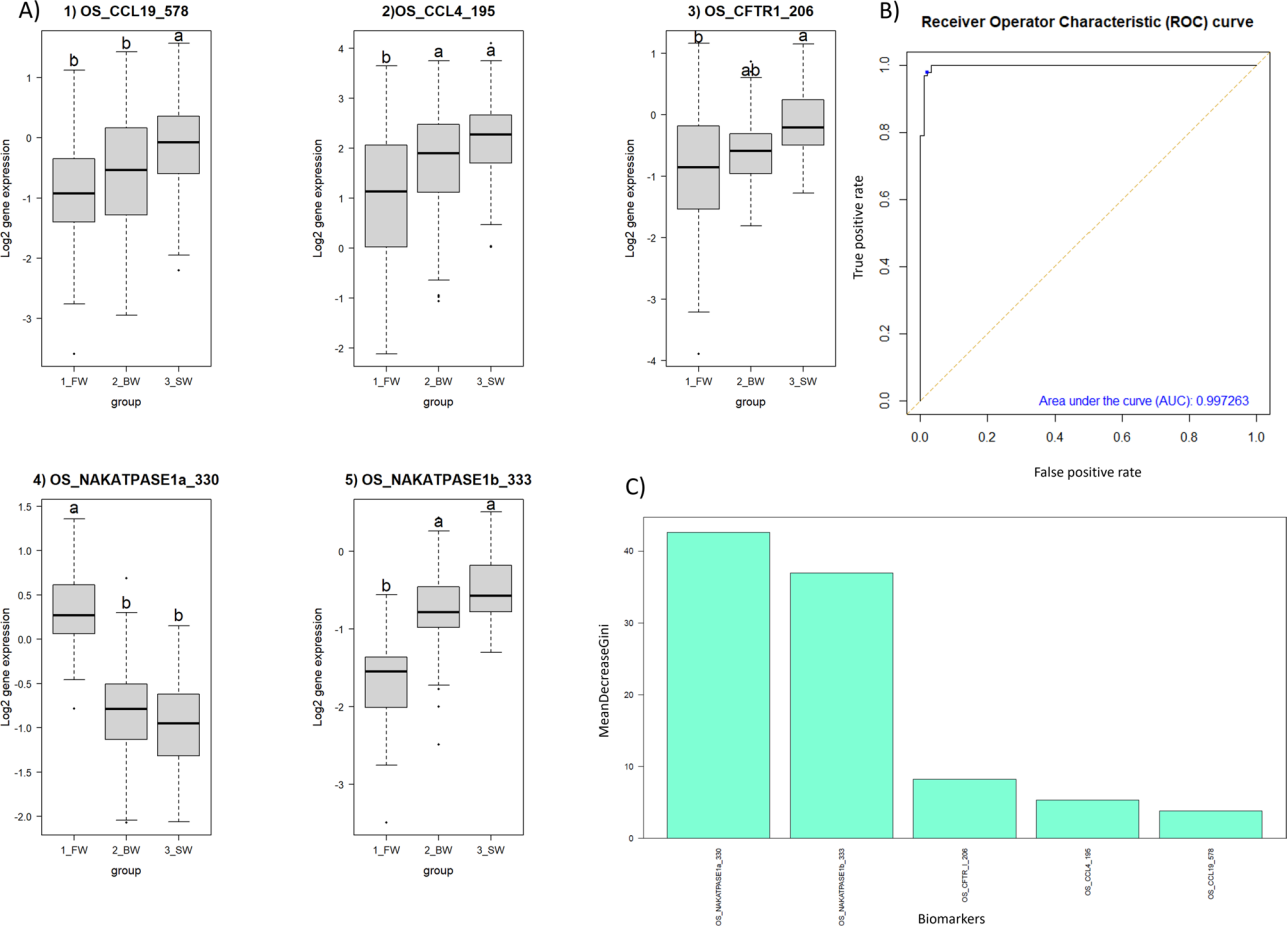
(**A**) The mRNA expression patterns of 5 selected salinity acclimation classifier biomarkers for RF analysis among freshwater brackish water and seawater groups. (**B**) Receiver Operating Characteristic ROC curve analysis of the RF model in the train dataset for salinity acclimation classifier. (**C**) The biomarker rank for salinity acclimation classifier based on mean decrease in accuracy and Gini scores. Data with different letters are significantly different among different smolt stages (*p* < 0.05; ANOVA/Tukey’s HSD tests).

A panel of five osmoregulation biomarkers provided optimal separation in classifying the fish into FW and SW treatments of the challenge trials, regardless of their smolt stage (Supplementary Table 5). The mRNA expression pattern of the salinity acclimation biomarkers among FW, BW and SW treatments is shown in Fig. 5A.

The mRNA expression of OS_CCL19_578 and OS_CFTR1_206 was significantly upregulated in fish transferred from freshwater to seawater for six days (*p* < 0.001; ANOVA/Tukey’s HSD tests). In contrast, exposure to brackish water did not affect the expression of these biomarkers. Two other biomarkers, OS_NAKATPASE1b_333 and OS_CCL4_195, were significantly upregulated in fish exposed to both brackish and seawater (*p* < 0.001; ANOVA/Tukey’s HSD tests). Conversely, the biomarker OS_NAKATPASE1a_330 was significantly downregulated in fish exposed to both brackish and seawater (*p* < 0.001; ANOVA/Tukey’s HSD tests).

The RF model for the salinity acclimation classifier on the training dataset (N = 195) showed an area under the ROC curve (AUC) of 99.7% (Fig. 5B). On the testing dataset (N = 93), the model showed high prediction accuracy of 100% for both FW and SW treatments (Table 2). The above five biomarkers were the most important salinity acclimation biomarkers in the model based on the Gini scores (Fig. 5C).

SW acclimated fish that were returned to freshwater could fully recover from seawater exposure within three days, as depicted by the reversal of expression of seawater expressed genes. RF-based modeling applied during the recovery trials indicated that over 95% of fish that had been acclimated to seawater for six days were classified as FW after three days back in freshwater (see Table 3).

Using the above five biomarkers, the PCA displayed visible separation of the FW and SW groups for both train and test datasets; however, BW was not separated from SW, while BW and FW fish showed some overlap in the ellipses (supplementary Fig. 2).

### Smolt stage classifier

**Fig. 6:**
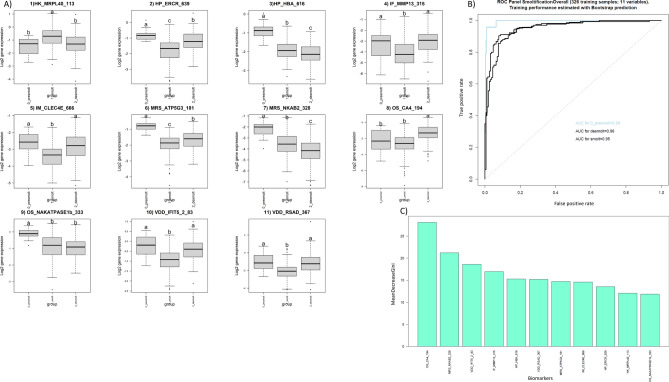
(**A**) The mRNA expression patterns of 11 selected smolt stage classifier biomarkers for RF analysis between smolt and de-smolt groups. (**B**) Receiver Operating Characteristic ROC curve analysis of the RF model in the train dataset for smoltification classifier. (**C**) The biomarker rank for smolt stage classifier based on mean decrease in accuracy and Gini scores. Data with different letters are significantly different among different smolt stages (*p* < 0.05; ANOVA/ Tukey’s HSD tests).

A panel of 11 biomarkers provided optimal separation in classifying fish into pre-smolt, smolt, and de-smolt stages across all salinity environments (Supplementary Table 6). Their mRNA expression patterns between the 3 stages is shown in Fig. 6A. Nine biomarkers were significantly downregulated, and one biomarker was significantly upregulated in smolt fish compared to pre-smolt fish (*p* < 0.05; ANOVA/ Tukey’s HSD tests). Seven biomarkers were significantly upregulated and three biomarkers were significantly downregulated in de-smolt fish compared to smolt fish (*p* < 0.05; ANOVA/ Tukey’s HSD tests). The biomarkers OS_CA4_194, and OS_NAKATPASE1b_333 did not show significant differences among pre-smolt, smolt and de-smolt stages, respectively (*p* > 0.05; ANOVA/ Tukey’s HSD tests).

The RF-based classifier for smolt stage applied to the training dataset (N = 326) showed an area under the ROC curve (AUC) of 99%, 96%, and 95%, for pre-smolt, smolt, and de-smolt stages, respectively (Fig. 6B). On the testing dataset (N = 156), the model showed a prediction accuracy of 96.5%, 87.7%, and 89.5% for pre-smolt, smolt, and de-smolt stages, respectively (Table 2). The biomarkers OS_CA4_194, MRS_NKAB1_328, and VDD_IFIT5-2_83 were the most important smolt status biomarkers in the model based on the Gini scores (Fig. 6C).

Based on the 11 selected smolt stage biomarkers, the PCA displayed a separation of three smolt stages for both the training and testing datasets with some overlap of the ellipses, particularly for smolt and de-smolt stages (Supplementary Fig. 3).

## Discussion

This study advances molecular diagnostics for sockeye salmon by successfully developing transcriptomic classifiers to detect thermal and hypoxic stress, salinity acclimation, and to differentiate smolt stages, providing a new powerful tool for transformative field applications. By bridging controlled multi-stressor trials with ecological insights, the Salmon Fit-Chips can monitor physiological health in wild juveniles and adults across lakes, rivers, estuaries, and ocean habitats, informing on the impacts of climate change.

The classifiers developed here demonstrated comparable predictive accuracy to those previously developed for coho salmon and Chinook salmon^22,42^, confirming the robustness and transferability of the Fit-Chip approach across salmonid species. However, the optimal biomarker combinations differed by species, highlighting the importance of species-specific classifier development. For example, while SERPIN and HSP family genes were key contributors to thermal stress detection in both coho salmon and sockeye salmon, the relative expression dynamics and recovery timelines varied between species, potentially reflecting differences in thermal tolerance thresholds or gene regulation.

The development of a highly accurate thermal stress classifier for sockeye salmon based on nine transcriptional biomarkers represents a critical advancement in molecular tools for assessing sub-lethal thermal exposure. Key genes such as HSP90a**,** SERPIN20**,** and SERPIN9 emerged as the most informative biomarkers, showing consistent and robust upregulation at elevated temperatures, particularly at 18 °C. These genes encode molecular chaperones and proteostasis regulators that are conserved indicators of thermal stress in vertebrates, reflecting disruptions to protein folding and cellular homeostasis^45,50^. Similarly, these genes were identified as the most robust biomarkers in thermal stress classifier of coho salmon^42^. Their graduated expression across 10 °C, 14 °C, and 18 °C, combined with the classifier’s AUC of 0.98 and predictive accuracy above 96% across all treatments highlights their specificity and sensitivity as diagnostic indicators. Furthermore, the persistence of stress signals following exposure to 18 °C, even after three days of recovery at 10 °C, underscores the potential of these biomarkers to capture not only chronic thermal stress but also residual physiological effects of recent thermal challenges. The persistence of thermal gene expression post-recovery observed here aligns with earlier work on coho salmon^42^ showing that transcriptional heat stress responses can remain elevated for up to 24–72 h following return to baseline conditions^50^, suggesting that Fit-Chips may capture a critical “stress history” window even after thermal exposures subside.

Such a tool is particularly timely given that wild sockeye salmon increasingly encounter thermally stressful conditions in their natural habitats, often exceeding physiological optima. Our previous studies on sockeye salmon, using earlier versions of the salmon Fit-Chip that lacked species-specific classifiers, revealed that juvenile and adult sockeye salmon often exhibit thermal stress signatures in response to seasonal and climate-driven warming^24,42,45^. In lakes such as Cultus Lake and Osoyoos Lake in British Columbia, juvenile sockeye salmon routinely experience high water temperature during summer and early fall stratification, triggering molecular heat stress responses detectable in gill tissue^24,42^. Similarly, adult sockeye salmon migrating upstream (e.g., the Fraser River and Columbia River) face high water temperatures that sharply decline their swimming power and migration success^51–53^. These conditions are projected to intensify with ongoing climate change, particularly in southern and interior populations. Therefore, the validated thermal stress classifier for sockeye salmon offers strong potential for field-based surveillance, enabling non-lethal, real-time monitoring of physiological vulnerability across habitats and life stages. By identifying individuals or populations under thermal duress, this tool can inform adaptive fisheries management, conservation prioritization, and habitat protection efforts tailored to increasingly dynamic thermal regimes.

The hypoxic stress classifier developed in this study, based on the expression of eight transcriptional biomarkers, provides a sensitive molecular tool for detecting sub-lethal dissolved oxygen stress in juvenile sockeye salmon. Among the top-ranked genes, HIF1A, RAMP1, and AURKB exhibited strong transcriptional responses to hypoxia. Hypoxia-inducible factors (HIFs) are key regulators of cellular responses to oxygen limitation across vertebrates, coordinating transcriptional programs that enable physiological and metabolic adaptation to low oxygen conditions^46,54^. In teleost fishes, the HIF gene family has undergone substantial diversification due to genome duplication events, resulting in multiple paralogs that may have distinct regulatory functions^55–57^.

HIF signaling is also regulated through complex mechanisms. In many vertebrates, hypoxic responses are controlled primarily at the post-translational level through oxygen-dependent stabilization of HIF proteins rather than large changes in transcript abundance^54,58^. Consistent with this, transcriptional responses of different HIF paralogs can vary across species and tissues^59^. Annotation of the most responsive transcript identified in this study (HP_HIF1A_262) against the *O. nerka* genome indicates that this sequence, designated as HIF1-alpha-like 2, most likely corresponds to HIF3A, based on high sequence similarity to HIF3A orthologs in related salmonids such as *O. mykiss* and *O. tshawytscha*. Recent comparative analyses of ray-finned fishes suggest that HIF3A-related paralogs are widespread and may be misannotated as HIF1-like genes due to their evolutionary origin following genome duplication events^57,60^. HIF3A is a comparatively understudied member of the HIF family and appears to function as a regulator of hypoxia signaling. Unlike HIF-1A and HIF-2A, which act as primary transcriptional activators, HIF-3A can produce multiple isoforms that modulate or fine-tune hypoxia-responsive pathways^57,59^. Evidence from fish models indicates that HIF3A may play an important role in hypoxia adaptation; for example, in zebrafish it contributes to hypoxia tolerance by regulating erythropoiesis and enhancing oxygen transport capacity under low oxygen conditions^61^.

In contrast, the bona fide HP_HIF1A_263 paralog examined in this study showed limited transcriptional regulation under hypoxia, consistent with the predominance of post-translational control of HIF-1A activity. The strong transcriptional response of the HIF1-alpha-like 2 (most likely HIF3A) transcript therefore suggests that this paralog may be particularly responsive to environmental oxygen stress in juvenile *O. nerka*. These findings highlight the importance of considering multiple HIF paralogs when developing transcriptional biomarkers of hypoxia exposure in salmonids, as functional specialization among duplicated genes may influence their diagnostic utility.

The bidirectional regulation pattern—four genes upregulated i.e., Hypoxia-inducible factor 1-alpha (HIF1A), Enolase (ENO2), Endothelin-converting enzyme 2 (ECE2), and Receptor activity-modifying protein 1 (RAMP1) and four downregulated i.e., Aurora kinase (AURK), Kinesin family member (KI1I), Ribonucleoside-diphosphate reductase subunit M2 (RR2R), and Non-SMC condensin II complex, subunit D3 (NCAPD3)—underscores the complexity of the hypoxic response, including transcriptional suppression of growth- and energy-related processes and activation of stress-adaptive pathways.

The moderate recovery rate observed—where ~ 40% of hypoxia-exposed fish shifted transcriptionally to a normoxic profile after 24 h, and ~ 50% after 72 h—suggests that some hypoxia signatures persist long after fish leave hypoxic environments, possibly due to sustained tissue-level metabolic disruption or slower transcriptomic reset in gill tissues. In contrast, full hypoxia recovery in coho salmon occurred in two days^42^. Our exposure trials targeted chronic rather than acute exposure, so it is unclear if shorter duration exposures would elicit a sustained response once fish enter normoxic conditions. These findings are important in light of growing evidence that juvenile and adult sockeye salmon regularly encounter low dissolved oxygen environments in natural habitats, especially under lake stratification or in eutrophic, warming water bodies. For example, in Cultus Lake**,** using an earlier version of Salmon Fit-Chips that did not have species-specific classifiers, we found that over one-third of juvenile sockeye caught at mid-depths during daylight hours exhibited transcriptomic signatures consistent with an hypoxia response during summer and early fall when the profundal zone became seasonally hypoxic because of thermal stratification and eutrophication, despite their diurnal migrations to normoxic surface waters to feed at night^24^. These data are consistent with a delay in hypoxia recovery in the field, as demonstrated here in a laboratory setting. Additionally, Brett^62^ and Davis^63^ reported that sockeye salmon are highly sensitive to dissolved oxygen levels below ~ 5 mg/L, especially during energetically demanding periods such as diel vertical migrations or smoltification. Given this ecological context, the hypoxic stress classifier validated here can be readily deployed in field-based surveillance programs to non-lethally monitor oxygen stress in wild fish. By identifying individuals under recent or chronic hypoxic exposure, the tool can guide conservation and habitat management—such as aeration interventions in lakes, flow regulation in rivers, or timing adjustments for smolt release from hatcheries—in a warming climate where stratification intensity and eutrophication are on the rise.

Proper osmotic acclimation is critical for outmigrating smolts entering seawater. The salinity acclimation classifier developed in this study achieved near-perfect classification accuracy in distinguishing FW and SW exposed juvenile sockeye salmon, underscoring the diagnostic strength of the five-gene panel in tracking osmoregulatory transitions. Key biomarkers such as Cystic fibrosis transmembrane conductance regulator I CFTR1 and Na/K ATPase α-1b (NAKATPASE1b) are well-known molecular indicators of ion transport and seawater acclimation, with expression tightly linked to increased ion exchange activity and chloride cell proliferation during smoltification and seawater entry^64,65^. Their upregulation in fish exposed to full-strength seawater, and the downregulation of Na/K ATPase α-1a (NAKATPASE1a), a freshwater-associated isoform, confirms their roles as reciprocal markers of osmoregulatory status. Interestingly, the limited separation between brackish water and seawater groups suggests that even moderate salinity triggers the full suite of transcriptional adjustments required for complete seawater acclimation. This pattern was observed in previous challenge experiments on Chinook salmon^22^ and coho salmon^42^—meaning that, to date, three salmon species have demonstrated this response.

In our recovery trials, over 95% of fish previously held in SW were classified as freshwater-acclimated after three days of return, suggesting rapid transcriptional reversibility of seawater-induced responses. In contrast, while reversibility of acclimation to FW was also demonstrated in coho salmon, only 36.4% of coho achieved full re-adaptation to FW after a three day re-exposure^42^. In general terms, reversibility reflects the plasticity of the salmonid gill and mirrors the physiological observations that smolts can re-acclimate to freshwater within days^66^. In the field, juvenile sockeye salmon frequently encounter salinity gradients during estuarine transition, which may be abrupt or prolonged depending on current, tide, and migration timing. The validated salinity acclimation classifier provides a powerful molecular tool to identify recent habitat usage in wild smolts or hatchery releases, potentially identifying fish that have recently moved between FW and SW habitats or into areas heavily influenced by FW. The integration of this signature of salinity adaptation with temperature and hypoxia classifiers can offer a holistic picture of both habitat usage and fish condition during one of the most physiologically demanding phases of the sockeye salmon life cycle.

The smolt stage classifier developed in this study demonstrates a high-resolution molecular approach to distinguish pre-smolt, smolt, and de-smolt stages in sockeye salmon, independent of salinity exposure. The RF-based model achieved excellent performance across smolt stages and high prediction accuracy on the testing dataset. Eleven biomarkers were found to optimally separate smolt stages, including Carbonic anhydrase 4 (CA4)**,** NKAB1**,** and Interferon-induced protein with tetratricopeptide repeats 5 (IFIT5-2)**,** which were among the top contributors based on Gini scores. Most notably, smolts were characterized by the downregulation of nine biomarkers compared to pre-smolts, including several biomarkers involved in immunity. Reduced immune competence and the downregulation of immune-related genes during the transition from parr to smolt are well-documented in salmonids (Johansson et al., 2016; Houde et al., 2019b; Krasnov et al. 2024). This is thought to reflect a trade-off, where energy and resources are redirected toward ion regulation and metabolic adaptation—processes essential for surviving in seawater. Given the high energetic demands of smoltification and the extensive physiological reprogramming it requires, both adaptive and innate immune responses are temporarily suppressed, increasing susceptibility to pathogens but enabling effective osmoregulatory changes^67–69^.

The transition to de-smolt was marked by a continued down-regulation of oxygen transport (HBA), and osmoregulatory adaptation (NKAB2) genes. At the same time, there was notable up-regulation of a gene involved in acid balance and ion regulation (CA4), and the reactivation of several pre-smolt markers involved in immunity and inflammation (e.g., IFIT5, RSAD, MMP13, CLEC4E). These changes suggest that during de-smoltification—when salmon transition from a seawater environment back to freshwater—certain genes and proteins are differentially regulated to support physiological re-adaptation^22,43^. This bidirectional expression pattern reflects the dynamic physiological remodelling associated with smoltification and subsequent loss of seawater readiness—a process that has critical implications for both wild and hatchery-reared salmonids.

Although sockeye salmon is not a species targeted for hatchery enhancement to support increased fisheries opportunities in Canada—partly because of their strong dependence on lake environments which requires at least one year in freshwater, and historically poor survival rates from fry releases^70,71^—many stocks in the southernmost distribution of North America are now threatened^42,72,73^. As a result, hatchery-based culture of sockeye salmon for rebuilding purposes has been increasingly adopted in both BC and Washington state with notable examples including efforts for Cultus Lake**,** Sakinaw Lake**,** Ozette Lake**,** and the Okanagan Lake system (ibid). Accurately identifying smolt status is essential to inform release timing that ensures readiness for the transitions to seawater environments. Hatcheries often rely on phenotypic cues such as body silvering, NKA activity, or fish size. However, such proxies can be unreliable and fail to detect fish that have already undergone de-smoltification—which can result in reduced post-release survival and increased vulnerability to additional stressors^43,74^. De-smoltification, in particular, is a growing concern under warming freshwater conditions and delayed releases, both of which can suppress seawater tolerance and impair body growth and osmoregulation upon ocean entry^42,64,75^. A Fit-Chip panel to track smoltification has been applied in Chinook salmon hatcheries for three years, and has revealed strong annual variance in the timing of full smolt readiness. The molecular classifier developed here for sockeye salmon offers a robust, high-throughput, and potentially non-lethal method to assess physiological smolt status with precision, making it an invaluable tool for hatcheries aiming to optimize release timing and reduce early marine mortality. Furthermore, the gene expression dynamics observed—such as the decline in smolt markers and reactivation of pre-smolt profiles during de-smoltification—are consistent with studies showing reversibility in osmoregulatory function in fish held too long in freshwater^76^. Given the interannual variability in environmental conditions and hatchery schedules, tools like the smolt stage Fit-Chip classifier can significantly enhance decision-making by providing individual-level, transcriptomic confirmation of seawater readiness. This capacity is increasingly important in the context of climate change, which can shift smolt timing windows and alter the relationship between environmental cues and internal developmental programs. These shifts in smolt windows will affect both cultured and wild fish. While cultured fish are reliant on hatchery release dates to time their migrations to the ocean, it is often assumed that wild fish will regulate their migration timing according to their smoltification schedule. Early data from application of Fit-Chips in our program suggest that it is not always the case (unpublished data). Hence, the smoltification panel can be applied to understand the dynamics of ocean entry behaviour of both hatchery and wild salmon, and with the addition of a panel to recognize imminent mortality, which was designed and validated for sockeye salmon from biomarkers discovered in Jeffries et al.^52^, and for Chinook salmon^22^ and coho salmon^42^, one can resolve the contribution of osmotic stress from ill-timed ocean entry on early marine survival.

Unlike previous studies in Chinook salmon (ocean-type) and coho salmon, where de-smolts exhibited heightened vulnerability to additional stressors such as elevated temperature and hypoxia— often resulting in increased and sometimes synergistic mortality^22,42,52^—the sockeye salmon examined in this study displayed a markedly different physiological response. Despite exposure to thermal and hypoxic stressors, sockeye salmon de-smolts were able to survive over a 6-day period in seawater with minimal, suggesting a greater resilience to compounding environmental challenges under the conditions tested. Because the experimental design used here mirrors that applied previously to other salmon species, these results provide a direct comparison of stress responses across species and highlight clear species-specific differences in resilience to environmental stressors. Such differences likely reflect variation in life-history strategies, ecological niches, and physiological adaptations among Pacific salmon^77,78^. It is also possible that the de-smolt individuals used in our trials retained partial seawater readiness, which may differ from the more advanced de-smolts examined in previous experiments with other species. Together, these findings reinforce developing species-specific molecular classifiers and reference datasets, as stress responses and tolerance thresholds cannot be assumed to be equivalent across salmonids. Recognizing these differences will improve the interpretation of genomic biomarker diagnostics and help inform management strategies, including hatchery release timing and habitat assessments for different salmon species.

## Conclusions

In conclusion, this study advances the Fit-Chip framework by developing and experimentally validating sockeye salmon–specific molecular classifiers capable of resolving transcriptional signatures of thermal stress, hypoxia, salinity acclimation, and smolt stage under controlled multi-stressor conditions. By focusing on gill tissue—an organ at the primary interface between fish and their environment—this work provides a robust molecular basis for assessing physiological responses to key environmental drivers associated with climate change. Although fish in this study were euthanized to obtain sufficient gill tissue for high-quality RNA analysis, the biomarkers and classifiers developed here are compatible with non-lethal sampling approaches, such as small gill biopsies commonly used in field studies. As such, these tools provide a foundation for future field applications aimed at monitoring the physiological condition of wild and hatchery populations. Integrating Fit-Chip classifiers with environmental monitoring and fish distribution data could ultimately enable more informed management decisions, including hatchery release timing and conservation strategies under changing environmental conditions. Future studies should prioritize field validation and explore integration with pathogen and microbiome data to further expand the diagnostic capacity of this platform.

## Supplementary Information

Supplementary Information 1: Canonical plots of the first two principal components of the 8 optimal biomarkers for hypoxic stress. Principal component analysis was performed on the 2/3 training set (A) and then tested to the 1/3 testing set (B). PCA was also applied on the 3 days recovery from hypoxia to normoxia set (C). Ellipses represent 95% confidence areas for the groups within treatments using the training set; centroids are represented by the largest point of the same colour. Arrows (left panel) represent loading vectors of the biomarkers using the training set.

Supplementary Information 2: Canonical plots of the first two principal components of the five optimal biomarkers for salinity acclimation. Principal component analysis was performed on the 2/3 training set (A) and then tested to the 1/3 testing set (B) using the freshwater, brackish water and seawater data. Ellipses represent 95% confidence areas for the groups within treatments using the training set; centroids are represented by the largest point of the same colour. Arrows (left panel) represent loading vectors of the biomarkers using the training set.

Supplementary Information 3: Canonical plots of the first two principal components of the 11 optimal biomarkers for smolt stage detection. Principal component analysis was performed on the 2/3 training set (A) and then tested to the 1/3 testing set (B). Ellipses represent 95% confidence areas for the groups within treatments using the training set; centroids are represented by the largest point of the same colour. Arrows (left panel) represent loading vectors of the biomarkers using the training set.

Supplementary Information 4.

Supplementary Information 5.

Supplementary Information 6.

Supplementary Information 7.

Supplementary Information 8.

Supplementary Information 9.

## Data Availability

The molecular data generated in this study are publicly available in the Zenodo repository athttps:10.5281/zenodo.19493158, and in the Open Government of Canada Portalat/pp https://open.canada.ca/data/en/dataset/d15fb0fe-b3c5-4cba-8f37-a2ae6facd903/p
