# Supplementary material for: Developing molecular classifiers to detect environmental stressors and smolt stages in sockeye salmon, Oncorhynchus nerka
Source: Sci Rep. 2026 Jun 4;16:25584. doi: 10.1038/s41598-026-49226-3 (PMC13478211; doi:10.1038/s41598-026-49226-3)
Supplement: Supplementary file 1 [file 41598_2026_49226_MOESM1_ESM.pdf]

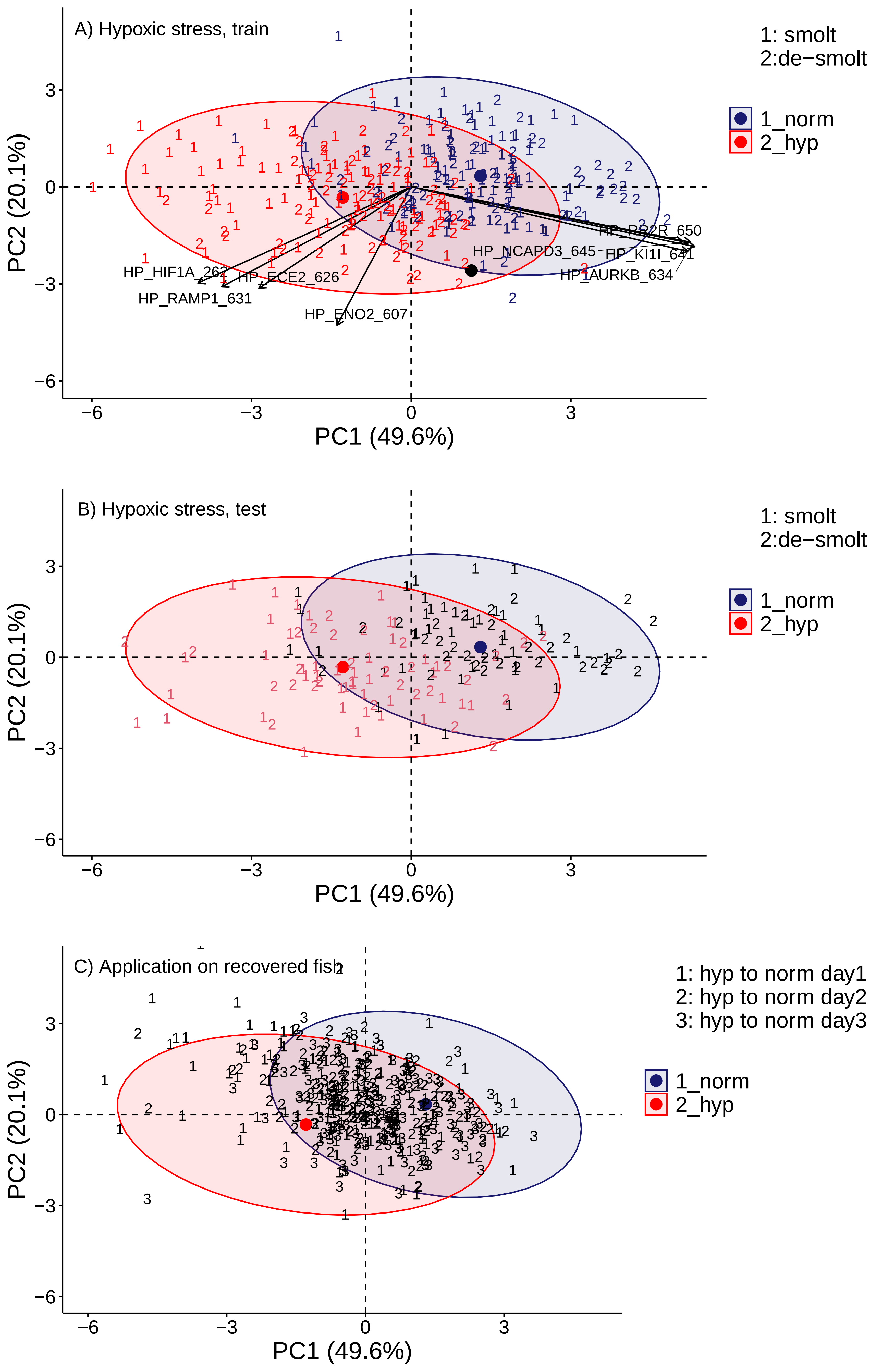
A) Hypoxic stress, train
B) Hypoxic stress, test
C) Application on recovered fish
1: smolt
2:de−smolt
1_norm
2_hyp
1: hyp to norm day1
2: hyp to norm day2
3: hyp to norm day3
PC1 (49.6%)
PC2 (20.1%)
HP_HIF1A_262
HP_RAMP1_631
HP_ECE2_626
HP_ENO2_607
HP_NCAPD3_645
HP_RR2R_650
HP_KI1I_641
HP_AURKB_634
