# Supplementary material for: Developing molecular classifiers to detect environmental stressors and smolt stages in sockeye salmon, Oncorhynchus nerka
Source: Sci Rep. 2026 Jun 4;16:25584. doi: 10.1038/s41598-026-49226-3 (PMC13478211; doi:10.1038/s41598-026-49226-3)
Supplement: Supplementary file 2 [file 41598_2026_49226_MOESM2_ESM.pdf]

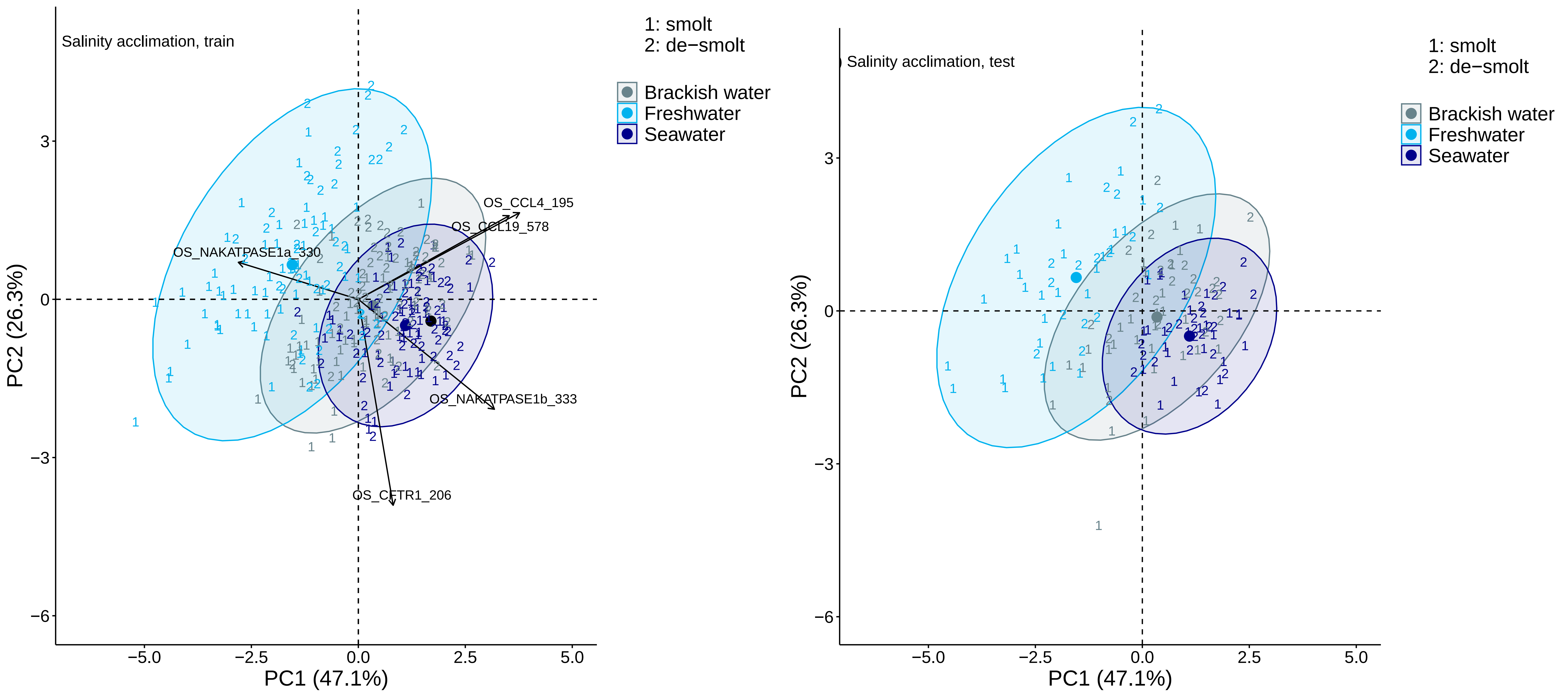

Salinity acclimation, train
1: smolt
2: de−smolt
Brackish water
Freshwater
Seawater
OS_CCL4_195
OS_CCL19_578
OS_NAKATPASE1a_330
OS_NAKATPASE1b_333
OS_CFTR1_206
PC1 (47.1%)
PC2 (26.3%)
Salinity acclimation, test
1: smolt
2: de−smolt
Brackish water
Freshwater
Seawater
PC1 (47.1%)
PC2 (26.3%)
