# Supplementary material for: Developing molecular classifiers to detect environmental stressors and smolt stages in sockeye salmon, Oncorhynchus nerka
Source: Sci Rep. 2026 Jun 4;16:25584. doi: 10.1038/s41598-026-49226-3 (PMC13478211; doi:10.1038/s41598-026-49226-3)
Supplement: Supplementary file 3 [file 41598_2026_49226_MOESM3_ESM.pdf]

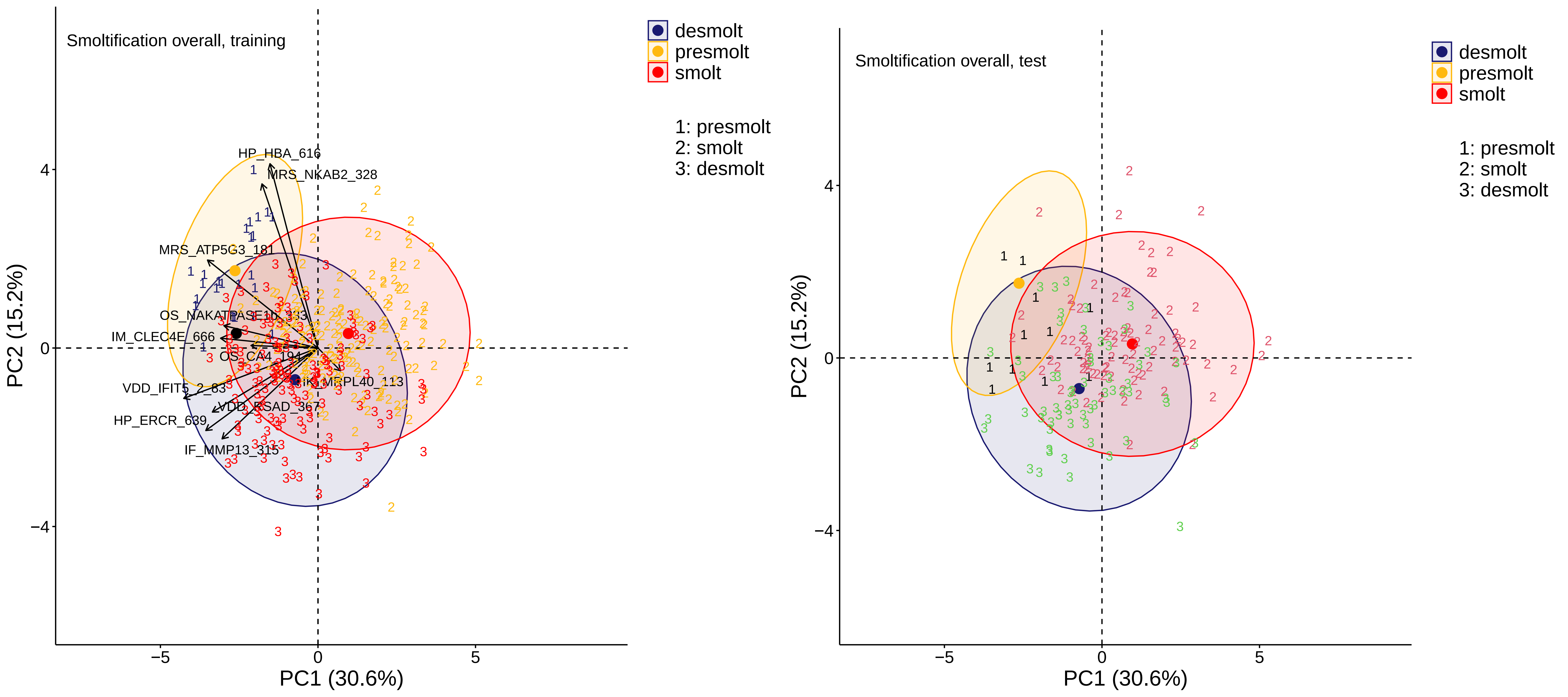

Smoltification overall, training
desmolt
presmolt
smolt
1: presmolt
2: smolt
3: desmolt
HP_HBA_616
MRS_NKAB2_328
MRS_ATP5G3_181
OS_NAKATPASE1b_343
IM_CLEC4E_666
OS_CA4_194
VDD_IFIT5_2_83
IK_MRPL40_113
VDD_RSAD_367
HP_ERCR_639
IF_MMP13_315
PC2 (15.2%)
PC1 (30.6%)
Smoltification overall, test
desmolt
presmolt
smolt
1: presmolt
2: smolt
3: desmolt
PC2 (15.2%)
PC1 (30.6%)
