# Supplementary material for: Developing molecular classifiers to detect environmental stressors and smolt stages in sockeye salmon, Oncorhynchus nerka
Source: Sci Rep. 2026 Jun 4;16:25584. doi: 10.1038/s41598-026-49226-3 (PMC13478211; doi:10.1038/s41598-026-49226-3)
Supplement: Supplementary file 4 [file 41598_2026_49226_MOESM4_ESM.docx]

Supplementary Table 1. The gene names, Fit-chips names, and the primers-probe information for the 96 biomarkers tested on sockeye Salmon (*Oncorhynchus nerka*)

| Gene name | Fit-chips name | Primers/Probes | Accession number |
| --- | --- | --- | --- |
| ***Housekeeping*** | | |  |
| S100-A13-like | HK_S100_10 | F-GTCAAGACTGGAGGCTCAGAG  R-GATCAAGCCCCAGAAGTGTTTG PAAGGTGATTCCCTCGCCGTCCGA | [XM_029663336.2](https://www.ncbi.nlm.nih.gov/entrez/viewer.fcgi?db=nucleotide&id=2727907693) |
| Mitochondrial ribosomal protein L40 | HK_MRPL40_113 | F-CCCAGTATGAGGCACCTGAAGG R-GTTAATGCTGCCACCCTCTCAC P-ACAACAACATCACCA | [XM_029678377.2](https://www.ncbi.nlm.nih.gov/entrez/viewer.fcgi?db=nucleotide&id=2727823301) |
| Centrosomal AT-AC splicing factor (cenatac) | HK_CCDC84_114 | F-GCTCATTTGAGGAGAAGGAGGATG  R-CTGGCGATGCTGTTCCTGAG  P-TTATCAAGCAGCAAGCC | [XM_029671520.2](https://www.ncbi.nlm.nih.gov/entrez/viewer.fcgi?db=nucleotide&id=2727808097) |
| ***Thermal stress*** | | |  |
| Heat shock 70 kDa protein | TM_HSP70_267 | F-TCAACGATCAGGTCGTGCAA  R-CGTCGCTGACCACCTTGAA  P-CCGACATGAAGCACTGG | [XM_029635774.2](https://www.ncbi.nlm.nih.gov/entrez/viewer.fcgi?db=nucleotide&id=2727797104)  [XM_065016558.1](https://www.ncbi.nlm.nih.gov/entrez/viewer.fcgi?db=nucleotide&id=2727797102) |
| Heat shock protein 90 alpha | TM_HSP90AA_269 | F-ATGACCCTCAGACACACTCCAA R-CCTCATCAATACCCAGTCCTAGCT  P-CGCATCTACAGAATGA | [XM_029631791.2](https://www.ncbi.nlm.nih.gov/entrez/viewer.fcgi?db=nucleotide&id=2727870120) |
| Heat shock protein 90 alpha like | TM_HSP90a_271 | F-TTGGATGACCCTCAGACACACT  R- CGTCAATACCCAGGCCTAGCT  P- CCGAATCTACCGGATGAT | [XM_029647220.2](https://www.ncbi.nlm.nih.gov/entrez/viewer.fcgi?db=nucleotide&id=2727820131) |
| Mitogen-activated protein kinase kinase kinase 14 | TM_MAP3K14_308 | F-GCTCCCTGGGTTCATGGAT R-GCCTCCCTTCAGCAGAGACA  P-CCAGCAATAGCTTATG | XM_029635839.2 |
| Peptidyl-prolyl cis-trans isomerase FKBP10-like (chr.26) | TM_FKBP10_2_582 | F-ACTATGAGAATGCCCCCATCAC R-CTCGTCCAGACCCTCAATCAC  P-CCTGGGAGCCAACAA | [XM_029635768.2](https://www.ncbi.nlm.nih.gov/entrez/viewer.fcgi?db=nucleotide&id=2727877939) |
| Peptidyl-prolyl cis-trans isomerase FKBP10-like (chr.23) | TM_FKBP10_4_583 | F-CCTGAAGAGATCATTGCTGACATG  R-GACGATGACCCCATCCTTGT  P-TCAGGAACCAGGACCG | [XM_029629529.2](https://www.ncbi.nlm.nih.gov/entrez/viewer.fcgi?db=nucleotide&id=2727866424) |
| Serpin H1 b (HSP47) (chr.11) | TM_SERPIN_20_379 | F-ACTATGACCACTCGAAGATCAACCT  R-CCCATTCGTTGATGGAGTTCA  P-AGGGACAAGAGGAGC | [XM_029672193.2](https://www.ncbi.nlm.nih.gov/entrez/viewer.fcgi?db=nucleotide&id=2727810294)  [XM_065024345.1](https://www.ncbi.nlm.nih.gov/entrez/viewer.fcgi?db=nucleotide&id=2727810296) |
| Serpin H1-like (HSP47) (chr.14) | TM_SERPIN_9_380 | F-GAGGTCAGCGACCCAAAGAC  R-GCCGTAGAGGCGGTTACTGAT  P-CGGAACGTCACATGGA | [XM_029681370.2](https://www.ncbi.nlm.nih.gov/entrez/viewer.fcgi?db=nucleotide&id=2727830509) |
| Heat shock protein HSP 90-alpha | GS_HSP90b_268 | F-TGGGCTACATGGCTGCCAAG R-TCCAAGGTGAACCCAGAGGAC  P-AGCACCTGGAGATCAA | [XM_029631791.2](https://www.ncbi.nlm.nih.gov/entrez/viewer.fcgi?db=nucleotide&id=2727870120) [XM_029647220.2](https://www.ncbi.nlm.nih.gov/entrez/viewer.fcgi?db=nucleotide&id=2727820131) |
| ***Hypoxia stress*** | | |  |
| Cytochrome C oxidase subunit 6B1 | HP_COX6B_216 | F-GCCCCGTGTGACTGGTATAAG  R-TCGTCCCATTTCTGGATCCA  P- TCTACAAATCACTGTGCCC | [XM_029629369.2](https://www.ncbi.nlm.nih.gov/entrez/viewer.fcgi?db=nucleotide&id=2727866177) |
| Hypoxia-inducible factor 1-alpha-like 2 (chr. 19) | HP_HIF1A_262 | F-AGAGGAGGCAGTGCTGTATTCAA  R-GGGACAAGGCCCTCCAAT  P-AGGGCCCTGACCATG | [XM_029620951.2](https://www.ncbi.nlm.nih.gov/entrez/viewer.fcgi?db=nucleotide&id=2727847576) |
| Hypoxia-inducible factor 1-alpha-like (chr. 14) | HP_HIF1A_263 | F-TGGCAAATCTGCCTACGAATT R-GCAGGCTCTTGGTCACATGA P-ATCATGCCCTGGACTC | [XM_029680742.2](https://www.ncbi.nlm.nih.gov/entrez/viewer.fcgi?db=nucleotide&id=2727829349) |
| Aldolase A, fructose-bisphosphate, b | HP_ALDOA1_687 | F-GGGAGTGCCATGTCTTATTCG  R-TGTTGCAGAGGGTCGAGTAGAG  P-ACCTCCTAAGAAGCCG | [XM_029629767.2](https://www.ncbi.nlm.nih.gov/entrez/viewer.fcgi?db=nucleotide&id=2727866727) |
| Anillin-like | HP_ANLN_633 | F-TGGTGAATGGATTTGGAGCTT  R-GGGTGGTTCCAATAGGACATGT  P-CGCTTCTTTGTCCTGGAG | [XM_029668234.2](https://www.ncbi.nlm.nih.gov/entrez/viewer.fcgi?db=nucleotide&id=2727918583)  [XM_029634072.2](https://www.ncbi.nlm.nih.gov/entrez/viewer.fcgi?db=nucleotide&id=2727875228) |
| Rho GTPase-activating protein 11A-like | HP_ARGR_648 | F-TGATAATTACTCCCAACTCCAAGAGA  R-CTTGCTGGAGCCATAACTTTGA  P-AACTTCCTTTGGAATCTG | [XM_029675336.2](https://www.ncbi.nlm.nih.gov/entrez/viewer.fcgi?db=nucleotide&id=2727817034) |
| Aurora kinase B-like | HP_AURKB_634 | F-GAAATGTGGTCGCTTCGATGA  R-CATCAGCCAACTCCTCCATGT  P-CAGCGCACTGCTAC | [XM_029644058.2](https://www.ncbi.nlm.nih.gov/entrez/viewer.fcgi?db=nucleotide&id=2727917080) |
| Cyclin dependent kinase inhibitor 1Bb | HP_CDKN1B_625 | F-CGTCCTCAGCGAAATGGAA  R-CCATTCGAATCTCCCGTTTAAT P-TCGATTTTTCAAGTCAAAC | [XM_029668384.2](https://www.ncbi.nlm.nih.gov/entrez/viewer.fcgi?db=nucleotide&id=2727918740) |
| Citron Rho-interacting kinase-like | HP_CIT_636 | F-GATCTCTAGGTTTCAGCGCAAGA  R-TGAGCTCCACATCCTTTTGGT  P-ACCTGGAGTCAGTTCT | [XM_029682958.2](https://www.ncbi.nlm.nih.gov/entrez/viewer.fcgi?db=nucleotide&id=2727835069) |
| Claspin-like | HP_CLSPN_637 | F-ATGCGGGCTGCCCTATC  R-CTCTTGAAGAACTGGTCGATGCT  P-CATGCCTGAGCCCAA | [XM_029663621.2](https://www.ncbi.nlm.nih.gov/entrez/viewer.fcgi?db=nucleotide&id=2727908057) |
| Endothelin-converting enzyme 2-like | HP_ECE2_626 | F-AGCAGACGCTGGGAGAGAAC  R-CACGCCTGGTACGCCTTATAG P-CTGACAACGGAGGCC | [XM_029629849.2](https://www.ncbi.nlm.nih.gov/entrez/viewer.fcgi?db=nucleotide&id=2727866870) |
| Enolase 1a | HP_ENO2_607 | F-GTCTGAGCGTCTGGCTAAGTACAAC  R-CCAGCGAAAACAGCCTTGTC  P-AGCTGCTCAGGATTG | [XM_065020953.1](https://www.ncbi.nlm.nih.gov/entrez/viewer.fcgi?db=nucleotide&id=2727911444) |
| Excision repair cross-complementation group 6-like | HP_ERCR_639 | F-TTGTATGGTCTCCACAGAGATGGT  R-GTCTTCCCTAAGCCCATGTCAT  P-TCAAGGAGGAATCCTAG | [XM_029670892.2](https://www.ncbi.nlm.nih.gov/entrez/viewer.fcgi?db=nucleotide&id=2727806828) |
| Glycogen phosphorylase muscle A | HP_GP_608 | F-CAGAACTGAAGGTCGCTTTCAA  R-CCCGTTTGTTTGCCACATTT  P-AGGCATCTCCATTTTA | [XM_029620923.2](https://www.ncbi.nlm.nih.gov/entrez/viewer.fcgi?db=nucleotide&id=2727847535) |
| Phosphorylase, glycogen; brain | HP_GPb_609 | F-CCTCTGCTTACTATTACTCCTGCTAGTCT  R-TGGGATTGGCATCTACAGTATACG P-TGACTCCACCATGTGC | [XM_029669643.2](https://www.ncbi.nlm.nih.gov/entrez/viewer.fcgi?db=nucleotide&id=2727804349) |
| Histone H2A.V | HP_H2F2_640 | F-CCCTCATCAAGGCCACCAT  R-CGATCAGGGACTTGTGGATGT  P-AGGCGGTGTGATCC | [XM_029669643.2](https://www.ncbi.nlm.nih.gov/entrez/viewer.fcgi?db=nucleotide&id=2727804349) |
| Hemoglobin subunit alpha-1 | HP_HBA_616 | F-CCTTGTGACCCTGGCTATTCA  R-GCAAGGAATTTATCCACAGCAAT  P-CACTCCCGAAGTGCA | [XM_029636000.2](https://www.ncbi.nlm.nih.gov/entrez/viewer.fcgi?db=nucleotide&id=2727878184)  [XM_029635952.2](https://www.ncbi.nlm.nih.gov/entrez/viewer.fcgi?db=nucleotide&id=2727878179)  [XM_029624473.2](https://www.ncbi.nlm.nih.gov/entrez/viewer.fcgi?db=nucleotide&id=2727860064) |
| Heme oxygenase 2b | HP_HMOX2_612 | F-AGACACTCTCAGCCCCACAGA  R-GGGTGTTCTCCGCCTTCTC  P-CTTTCTGAACTGCTGGCT | [XM_065010601.1](https://www.ncbi.nlm.nih.gov/entrez/viewer.fcgi?db=nucleotide&id=2727879625) |
| Kinesin family member 15 | HP_KI1I_641 | F-CAGGCAGGTCTTCTCCAAGCT  R-AGTTTGGATGATAGCCTCCTTCTG  P-CACAGGATCAGACTGC | [XM_029675505.2](https://www.ncbi.nlm.nih.gov/entrez/viewer.fcgi?db=nucleotide&id=2727817411) |
| Kinesin family member 4 or Chromosome-associated kinesin KIF4-like | HP_KIF4_635 | F-GAATCTGCAGCACGTCATCCT  R-CAGCCAACGCCTCAATGG  P-CAGCTCCAGGATGAGAGT | [XM_029620685.2](https://www.ncbi.nlm.nih.gov/entrez/viewer.fcgi?db=nucleotide&id=2727847191)   [XM_029670562.2](https://www.ncbi.nlm.nih.gov/entrez/viewer.fcgi?db=nucleotide&id=2727806075) |
| Malignant fibrous histiocytoma-amplified sequence 1 homolog | HP_MFHAS1_629 | F-CCGAGGCCTGGGTGAAC  R-TCAGCTGCTCCACAGAGAAGAA  P-TCAGTGGCTGCTAGTC | [XM_029628332.2](https://www.ncbi.nlm.nih.gov/entrez/viewer.fcgi?db=nucleotide&id=2727864324) |
| Non-SMC condensin II complex, subunit D3 | HP_NCAPD3_645 | F-TCACAGGTCCAGAAGAAAGTGTTC  R-GCGAGCGCTTCTGTTCCA  P-TCGAGAGCATGATTCC | [XM_029671326.2](https://www.ncbi.nlm.nih.gov/entrez/viewer.fcgi?db=nucleotide&id=2727807723) |
| Kinetochore protein NDC80 homolog | HP_NDC80_644 | F-CCGGCTCCAACACATCTTGT  R-CTTCTCCCAGTTGATCCTCTCAA  P-CACCCAGAAGTTCACTC | [XM_029637104.2](https://www.ncbi.nlm.nih.gov/entrez/viewer.fcgi?db=nucleotide&id=2727881750)  [XM_029637103.2](https://www.ncbi.nlm.nih.gov/entrez/viewer.fcgi?db=nucleotide&id=2727881749) |
| Neuroglobin-1 | HP_NGB1_613 | F-GCCTCTCCAGCCCTGAGTTC  R-GGAGGTTGTCCAGGTGACTGA  P-TGGACCATGTCACAAAG | [XM_065004225.1](https://www.ncbi.nlm.nih.gov/entrez/viewer.fcgi?db=nucleotide&id=2727844890) |
| Peptidylglycine alpha-amidating monooxygenase | HP_PAM_630 | F-TGTAGGAGATGCAGCCACAGA  R-TCTTAACAGAGCGATGTTCAGCTT P-TGCTAAAGTTCTCCTCTGAC | [XM_029646549.2](https://www.ncbi.nlm.nih.gov/entrez/viewer.fcgi?db=nucleotide&id=2727894384) |
| Kinesin-like protein KIF2C | HP_PGK_642 | F-CGGCCAAACTGGAAGTGGTA  R-TTCTGGCTCTTCCCTGAAAAGT  P-AACTCACACAATGGGAG | [XM_029623713.1](https://www.ncbi.nlm.nih.gov/entrez/viewer.fcgi?db=nucleotide&id=1695908741) |
| Receptor activity-modifying protein 1-like | HP_RAMP1_631 | F-CGAACCAAGTGGTGCAAGACT  R-CCGGACATGCCTGGAAGA  P-CTTCATCCAGATCCATTC | [XM_029629605.2](https://www.ncbi.nlm.nih.gov/entrez/viewer.fcgi?db=nucleotide&id=2727866519) |
| Ribonucleoside-diphosphate reductase subunit M2 | HP_RR2R_650 | F-TGCTGCTAGTGATGGCATTGT  R-TTTGGAAACCATAGAAGCATCTTG  P-ATTTACACAGGAAGTCCAGG | [XM_029631062.2](https://www.ncbi.nlm.nih.gov/entrez/viewer.fcgi?db=nucleotide&id=2727869119)  [XM_029677246.2](https://www.ncbi.nlm.nih.gov/entrez/viewer.fcgi?db=nucleotide&id=2727821830) |
| Structural maintenance of chromosomes protein 4-like | HP_SM4M_651 | F-AGCGTGTGGCTGAGTTGGAT  R-GATGTTGAAGCCAGCCATGA  P-ATCACCACAGAGAGAGACA | [XM_029672711.2](https://www.ncbi.nlm.nih.gov/entrez/viewer.fcgi?db=nucleotide&id=2727811175) |
| Transcription factor SOX-5-like | HP_SOX5_632 | F-GAGAGTACAAGGCCATTATGAGGAA  R-GCCCGCCGAGGACAAG P-CGGCAGGAGATGAG | [XM_065021683.1](https://www.ncbi.nlm.nih.gov/entrez/viewer.fcgi?db=nucleotide&id=2727914640) [XM_065008189.1](https://www.ncbi.nlm.nih.gov/entrez/viewer.fcgi?db=nucleotide&id=2727867409) |
| Tonsoku-like protein | HP_TOSO_652 | F-CCAATGCTTGCACCAATCAG  R-TCTGCCTCGCTATGTGGTACTG P-TGCGGGTCAGGGTT | [XM_029675015.2](https://www.ncbi.nlm.nih.gov/entrez/viewer.fcgi?db=nucleotide&id=2727816599) |
| Atos homolog A | HP_FAM214A_627 | F-GATGAGGAGTGTGTCTGTCAGTTCTC  R-GAGCCTCAGGGCAACACTCT  P-CCAGCGGAGCACCT | [XM_029648392.2](https://www.ncbi.nlm.nih.gov/entrez/viewer.fcgi?db=nucleotide&id=2727793350) |
| Insulin-like growth factor binding protein-1 | HP_IGFBP1B_284 | F-AGATAACCAGCTCTCAGCAGGAA  R-ATGTTTGTCACAGTTGGGTAGGTAGA  P-TAGGAGAGAAGTTCACCAAC | [XM_029642821.2](https://www.ncbi.nlm.nih.gov/entrez/viewer.fcgi?db=nucleotide&id=2727920618) |
| Phosphoglycerate kinase | HP_PgK3_343 | F-GGCAAAGTGCTCCCTAAGTTTC  R-TAGAGAGCAGGGCTGGTGCTA  P-CACCCTGCGCTTGT | [XM_029671204.2](https://www.ncbi.nlm.nih.gov/entrez/viewer.fcgi?db=nucleotide&id=2727807415) |
| ***Imminent mortality (IM)*** | | |  |
| Arrestin domain containing 2 | IM_ARRDC2_663 | F-AAGAAAGCCAAGGCGTGAGTAA  R-TCGGTTGCCAGGGTTAGC  P-TGGAGGACAAATCGGA | [XM_029635359.2](https://www.ncbi.nlm.nih.gov/entrez/viewer.fcgi?db=nucleotide&id=2727880932) |
| C-type lectin domain family 4, member E-like | IM_CLEC4E_666 | F-CCTGAGGGCTGGATTCATGT  R-TCGGCCAGTCCATCTTGTC  P-TGAGAAATGTTACTCCTTCAGT | [XM_029680132.2](https://www.ncbi.nlm.nih.gov/entrez/viewer.fcgi?db=nucleotide&id=2727828535) |
| Glutamate-ammonia ligase (glutamine synthetase) | IM_GLUL_670 | F-GTTCCAGGTTGGCCCTTGT  R-CCTAGCTGCCCAAAGGTGATC  P-AAGGCATCAGCATGGG | [XM_029623579.2](https://www.ncbi.nlm.nih.gov/entrez/viewer.fcgi?db=nucleotide&id=2727856823) |
| Gutathione peroxidase 3-like | IM_GPX3_628 | F- AGGCCAGTCCTTCAGTGCAT  R-GGCAGGACCAGGAGGTAACA  P-TGGGCCTGGTAACC | [XM_029644116.2](https://www.ncbi.nlm.nih.gov/entrez/viewer.fcgi?db=nucleotide&id=2727899094) |
| H1 histone family, member 0 | IM_H1F0_671 | F-CCAATGGACGTCAGCAAGATT  R-AGCATAGAGTCCGCATTTGGA  P-TCATGTGATGCGTAATGG | [XM_029629431.2](https://www.ncbi.nlm.nih.gov/entrez/viewer.fcgi?db=nucleotide&id=2727866259) |
| Heat shock protein 90-beta-like | IM_HSP90ab_270 | F-GACACGGTGTTGGGTTGGTT  R-TTGCAGTCAACTCTCCATGCA  P-TCATGTGCAACATAACAT | [XM_029631042.2](https://www.ncbi.nlm.nih.gov/entrez/viewer.fcgi?db=nucleotide&id=2727869078) |
| IQ motif containing GTPase activating protein 1 | IM_IQGAP1_675 | F-GAGGGTGTGGCTGTGATGAA  R-CAGGAAGATGAGCAGGTTGACA  P-CTCTTCGACAGGGCC | [XM_065012171.1](https://www.ncbi.nlm.nih.gov/entrez/viewer.fcgi?db=nucleotide&id=2727885427) |
| L-lactate dehydrogenase B-A chain-like | IM_LDH1_306 | F-GTCACTGCTCCCATTTTACACTCTAG  R-CCCAAACTCCCTCCCAGATAAC  P-CTGTTCTTAGCTTCCC | [XM_065022322.1](https://www.ncbi.nlm.nih.gov/entrez/viewer.fcgi?db=nucleotide&id=2727916957) |
| Nuclear protein 1 | IM_NUPR1_677 | F-GGAAGCCAGCGACAATACCA  R-GGGTTAGCCGTCCGATTTG  P-CACGAGCGCAAGCT | [XM_029635359.2](https://www.ncbi.nlm.nih.gov/entrez/viewer.fcgi?db=nucleotide&id=2727880932) [XM_065003174.1](https://www.ncbi.nlm.nih.gov/entrez/viewer.fcgi?db=nucleotide&id=2727841394) |
| Ornithine decarboxylase 1 | IM_ODC1_678 | F-CCAGAAGGCTCCCTGTTTCA  R-GCAGCCATTTCCTGGAGAAG  P-ACAACCCAATCTCA | [XM_029687914.2](https://www.ncbi.nlm.nih.gov/entrez/viewer.fcgi?db=nucleotide&id=2727844616) |
| Transgelin-2-like | IM_TAGLN3_681 | F-TGGCTCAAGGACGGATGTG  R-GGATCTTCCTGATGGGCTTGT  P-TGTGTGAACTGATCAACAG | [XM_029627966.2](https://www.ncbi.nlm.nih.gov/entrez/viewer.fcgi?db=nucleotide&id=2727863843) |
| ***Mortality related signature (MRS)*** | | |  |
| ATP synthase F(0) complex subunit C3, mitochondrial-like | MRS_ATP5G3_181 | F-GGAACGCCACCATGAGACA  R-CGCCATCCTGGGCTTTG  P-AGCCCCATTGCCTC | [XM_029635600.2](https://www.ncbi.nlm.nih.gov/entrez/viewer.fcgi?db=nucleotide&id=2727877750) |
| Complement component 7b | MRS_C7_189 | F-GATGCTGACCACATCAAACTGC  R-ACCTCTGTCCAGCTCTGTGTC  P-AACTACCAGACAGTGCTG | [XM_029651406.2](https://www.ncbi.nlm.nih.gov/entrez/viewer.fcgi?db=nucleotide&id=2727895406) |
| FYN-T-binding protein b | MRS_FYB_241 | F-TGCAGATGAGCTTGTTGTCTACAG  R-GCAGTAAAGATCTGCCGTTGAGA  P-CTCAACGATGACATCCACAGTCTCCCC | [XM_029655484.2](https://www.ncbi.nlm.nih.gov/entrez/viewer.fcgi?db=nucleotide&id=2727896013) |
| Histone acetyltransferase KAT5-like | MRS_HTATIP_272 | F-CTTGTAACAGTTCGACATGGCTTATT  R-TGGTGAAGCATTTCTGTATGTCAA  P-TCTGTACTGAGCATCCCCGCACATTACA | [XM_029620764.2](https://www.ncbi.nlm.nih.gov/entrez/viewer.fcgi?db=nucleotide&id=2727847288) |
| Uncharacterized protein LOC794943 homolog (zgc:194312) | MRS_IRF1_299 | F-CAAACCGCAAGAGTTCCTCATT  R-AGTTTGGTTGTGTTTTTGCATGTAG  P-CTGGCGCAGCAGATA | [XM_029667256.2](https://www.ncbi.nlm.nih.gov/entrez/viewer.fcgi?db=nucleotide&id=2727915697) |
| Keratin, type II cytoskeletal 8-like | MRS_KRT2_8_305 | F-CGATTGAGCGGCTGGATAA  R-GCATTGTTTACCTTTGACTTGAATTG  P-CCCCCTTCTCTACTCTCTTGCTCACCATTC | [XM_029622780.2](https://www.ncbi.nlm.nih.gov/entrez/viewer.fcgi?db=nucleotide&id=2727855811) |
| Sodium/potassium-transporting ATPase subunit beta-233-like | MRS_NKAB1_328 | F-CGTCAAGCTGAACAGGATCGT  R-CCTCAGGGATGCTTTCATTGGA P-CCTTGGCCTGAAGTTG | [XM_065023626.1](https://www.ncbi.nlm.nih.gov/entrez/viewer.fcgi?db=nucleotide&id=2727849470) |
| *Oncorhynchus mykiss* G-protein (P-ras) mRNA, complete cds | MRS_PRAS_350 | F-GCAGGATGAGCAGAGGAAGAA  R-GGCCTGGGCAATGTAACACT  P-CCCCCTAAAGATGCAG | [NM_001124705.1](https://www.ncbi.nlm.nih.gov/entrez/viewer.fcgi?db=nucleotide&id=185132100) |
| Secretogranin-2b | MRS_SCG_377 | F-GGATGTGAAGAATCCAACACTGAT  R-ACACCACTTCAAACTAGCCATACATT  P-CGGCTGTATGTGCACTG | [XM_029651224.2](https://www.ncbi.nlm.nih.gov/entrez/viewer.fcgi?db=nucleotide&id=2727830613) |
| ***Smoltification/Osmoregulation*** | | |  |
| Carbonic anhydrase 4-like | OS_CA4_194 | F-CGTTACCTTGGCTCCCTGACT  R-CTTGAACACGGTCCAAACCA  P-TCCAAATTGCAACGAGG | [XM_029643189.2](https://www.ncbi.nlm.nih.gov/entrez/viewer.fcgi?db=nucleotide&id=2727921159) |
| Chemokine (C-C motif) ligand 19a, tandem duplicate 1 | OS_CCL19_578 | F-ACCTGGGTTACAGACCTGATGAA  R-TGGTTTCGTGGCATTTCTTG  P-CTCATGGACCGCCTCA | [XM_029637092.2](https://www.ncbi.nlm.nih.gov/entrez/viewer.fcgi?db=nucleotide&id=2727881668) |
| Chemokine (C-C motif) ligand 19a, tandem duplicate 2 | OS_CCL4_195 | F-TCTCTTCATTGCAACAATCTGCTT  R-ACAGCAGTCCACGGGTACCT  P-CTACGCAGCAGCATT | [XM_029637093.2](https://www.ncbi.nlm.nih.gov/entrez/viewer.fcgi?db=nucleotide&id=2727881325) |
| CCAAT/enhancer binding protein (C/EBP), beta | OS_CEBPB_665 | F-AACTGGCCGCAGAGAATGAC  R-AAGTTACGCAGAGTGGCAAGCT  P-TTTACAAAAACGCGTGGAGC | [XM_029664963.2](https://www.ncbi.nlm.nih.gov/entrez/viewer.fcgi?db=nucleotide&id=2727909697) |
| Cystic fibrosis transmembrane conductance regulator-like | OS_CFTR1_206 | F-GAGCTGTCAGAGAGGAAGTTCTCA  R-GCAGCGACTCTTCAACCTGAT  P-TGGTGCCCGAGGAC | [XM_029634857.2](https://www.ncbi.nlm.nih.gov/entrez/viewer.fcgi?db=nucleotide&id=2727876995) |
| CD209 antigen-like protein E | OS_CLEC4M_211 | F-CGTGGCACTGAACTCTTATGCA  R-GGTACAGCCACTGTCACACTTGA  P-TGAGGGCTTCGATGAC | [XM_029628928.1](https://www.ncbi.nlm.nih.gov/entrez/viewer.fcgi?db=nucleotide&id=1695918515) |
| Elongation factor 2b | OS_EEF2_580 | F-CCACCAAGATTTCCCAGATTGT  R-TGCAGGAATTCCCTCCTTGA  P-TGACACACGTAAACGC | [XM_029685980.1](https://www.ncbi.nlm.nih.gov/entrez/viewer.fcgi?db=nucleotide&id=1695890634) |
| Peptidyl-prolyl cis-trans isomerase FKBP5-like | OS_FKBP5_584 | F-GGGCGTTCCTCTGGGTGTA  R-GCATGCAGCATTCTCCTTTCT  P-ACAGGGCCATGGAGA | [XM_065001752.1](https://www.ncbi.nlm.nih.gov/entrez/viewer.fcgi?db=nucleotide&id=2727835001) |
| Hemoglobin subunit  alpha-1 | OS_HBA_254 | F-GCCCTGGCTGACAAATACAGA  R-GAGCAGGAACTGGAGTCCAATG  P-ACCATCATGAAAGTCC | [XM_029636000.2](https://www.ncbi.nlm.nih.gov/entrez/viewer.fcgi?db=nucleotide&id=2727878184) |
| Na/K ATPase α-1a (freshwater) | OS_NAKATPASE1a_330 | F-TGGAATCAAGGTTATCATGGTCACT  R-CCCACACCCTTGGCAATG  P-ATCATCCCATCACTGCGA | [XM_029648252.2](https://www.ncbi.nlm.nih.gov/entrez/viewer.fcgi?db=nucleotide&id=2727901583) |
| Na/K ATPase α-1b (saltwater) | OS_NAKATPASE1b_333 | F-GCCTGGTGAAGAATCTTGAAGCT  R-GAGTCAGGGTTCCGGTCTTG  P-CCTCCACCATTTGCTCA | [XM_065018947.1](https://www.ncbi.nlm.nih.gov/entrez/viewer.fcgi?db=nucleotide&id=2727901579) |
| Nictoinaminde phosphoribosyltransferase-like | OS_NAMPT_593 | F-CCAACACACTACGCAGAAAGGA  R-CCTTGGTGGTCAGTGCTGTACA  P-TGGATCCCTTCTACCTCC | [XM_029644710.2](https://www.ncbi.nlm.nih.gov/entrez/viewer.fcgi?db=nucleotide&id=2727916873) |
| NADH dehydrogenase 1 beta subcomplex subunit 2 mitochondrial-like | OS_NDUFB2_322 | F-GCTTCTTACACGTGGACCTCAGA  R-GCCTGTACTGGGCCTCTATGTG  P-CGAAAAGCTGGTGGTGGA | [XM_029646938.2](https://www.ncbi.nlm.nih.gov/entrez/viewer.fcgi?db=nucleotide&id=2727867940) |
| Regulation of G protein signalling 21-like | OS_RGS21_597 | F-TCCCGACTACAGCGCAGAT  R-TCCTCAGGGCTAAGTCGTTCA  P-TTCCCAATCCCCC | [XM_029624215.2](https://www.ncbi.nlm.nih.gov/entrez/viewer.fcgi?db=nucleotide&id=2727857778) |
| Rhesus blood group, D antigen (rhd) | OS_RHAG_599 | F-GGTAGCTATGGCGATTGGATTT  R-AATGTATCGGGATCCCAAGGT  P-TGCTGCACTGATATC | [XM_029688294.2](https://www.ncbi.nlm.nih.gov/entrez/viewer.fcgi?db=nucleotide&id=2727845104) |
| ***Chloride levels*** | | |  |
| H-2 class II histocompatibility antigen, I-E beta chain | CL_H2EB1_672 | F-CAGTTGAGCCCCATGTCAGA  R-TCAGCATGGCAGGGTGTCT  P-TGAGCTCAGTGACTCC | [XM_065001859.1](https://www.ncbi.nlm.nih.gov/entrez/viewer.fcgi?db=nucleotide&id=2727835653) |
| HLA class II histocompatibility antigen gamma chain-like | CL_HLA21-G1_684 | F-CCAGGACGTTATCCTCCCAAT  R-GAGAAGACACGCCAGCACTGT  P-AGGGCCTCTAACAGC | [XM_065018523.1](https://www.ncbi.nlm.nih.gov/entrez/viewer.fcgi?db=nucleotide&id=2727899887) |
| Proteasome 20S subunit beta 8A (psmb8a) | CL_PSMB8_680 | F-CTGGTTGTGGTAGCAGCTATGC  R-CGCCTCCTCTACCGTCATGT  P-TACGGAGTGATGGACAGC | [XM_029663462.2](https://www.ncbi.nlm.nih.gov/entrez/viewer.fcgi?db=nucleotide&id=2727907883) |
| ***Viral Disease Development*** | | |  |
| Galectin-3-binding protein A-like | VDD_GAL3_72 | F-TTGTAGCGCCTGTTGTAATCATATC R-TACACTGCTGAGGCCATGGA  P-CTTGGCGTGGTGGC | [XM_065010682.1](https://www.ncbi.nlm.nih.gov/entrez/viewer.fcgi?db=nucleotide&id=2727879804) |
| Probable E3 ubiquitin-protein ligase HERC6 | VDD_HERC6_77 | F-AGGGACAACTTGGTAGACAGAAGAA R-TGACGCACACACAGCTACAGAGT  P-CAGTGGTCTCTGTGGCT | [XM_065003883.1](https://www.ncbi.nlm.nih.gov/entrez/viewer.fcgi?db=nucleotide&id=2727843245) |
| Interferon-induced protein 44-like | VDD_IFI44A_81 | F-CGGAGTCCAGAGCAGCCTACT R-TCCAGTGGTCTCCCCATCTC  P-CGCTGGTCCTGTGTGA | [XM_029633749.2](https://www.ncbi.nlm.nih.gov/entrez/viewer.fcgi?db=nucleotide&id=2727874376) |
| Interferon-induced protein with tetratricopeptide repeats 5-like | VDD_IFIT5-2_83 | F-CCGTCAATGAGTCCCTACACATT R-CACAGGCCAATTTGGTGATG P-CTGTCTCCAAACTCCCA | [XM_029684675.2](https://www.ncbi.nlm.nih.gov/entrez/viewer.fcgi?db=nucleotide&id=2727838182) |
| Interferon-induced GTP-binding protein Mx3 | VDD_MX_86 | F-AGATGATGCTGCACCTCAAGTC R-CTGCAGCTGGGAAGCAAAC P-ATTCCCATGGTGATCCGCTACCTGG | [XM_029683585.2](https://www.ncbi.nlm.nih.gov/entrez/viewer.fcgi?db=nucleotide&id=2727835878) |
| NFX1-type zinc finger-containing protein 1-like | VDD_NFX_87 | F-CCACTTGCCAGAGCATGGT R-CGTAACTGCCCAGAGTGCAAT P-TGCTCCACCGATCG | [XM_029684877.2](https://www.ncbi.nlm.nih.gov/entrez/viewer.fcgi?db=nucleotide&id=2727852883) |
| Radical S-adenosyl methionine domaine-containing 2 | VDD_RSAD_367 | F-GGGAAATTAGTCCAATACTGCAAAC R-GCCATTGCTGACAATACTGACACT P-CGACCTCCAGCTCC | [XM_029675046.2](https://www.ncbi.nlm.nih.gov/entrez/viewer.fcgi?db=nucleotide&id=2727816636) |
| Tripartite motif-containing protein 16-like | VDD_TRIM1_409 | F-CATGATGTCTGGTGTTGATGTATATTG R-GAGACAGAGAACCAACTGAGAAAACATA P-TTGTCATTCAGAACCATTG | [XM_065002886.1](https://www.ncbi.nlm.nih.gov/entrez/viewer.fcgi?db=nucleotide&id=2727839930) |
| ***Inflammation*** |  |  |  |
| Interleukin 17d | IF_IL17D_294 | F-CAACAGAAGTGCGAACGATG  R-GATGCCACATCGCATAACAG P-TGGTCGAGTATCTTTCGTGTGTTTGC | [XM_029662688.2](https://www.ncbi.nlm.nih.gov/entrez/viewer.fcgi?db=nucleotide&id=2727801938) |
| Collagenase 3-like | IF_MMP13_315 | F-GCCAGCGGAGCAGGAA R-AGTCACCTGGAGGCCAAAGA P-TCAGCGAGATGCAAAG | [XM_029671027.2](https://www.ncbi.nlm.nih.gov/entrez/viewer.fcgi?db=nucleotide&id=2727807033) |
| ***Saprolegnia*** |  |  |  |
| *Saprolegnia spp*. | sasp_480 | F-GCATTCAAGTTTGTGGGAAC R-CGGAAACCTTGTTACGACTTC P-TCCTTAACCTCGCCATTTAGAGGAAGG | [FJ794908.1](https://www.ncbi.nlm.nih.gov/entrez/viewer.fcgi?db=nucleotide&id=226819003) |
| *Saprolegnia parasitica* | sapa_481 | F-AGAGCAAATCGCGGTAGTTT R-AGAAATGCACCAGCATACCA P-TGCCTTGTACTTTGACAACAGACTCGC | [PQ309566.1](https://www.ncbi.nlm.nih.gov/entrez/viewer.fcgi?db=nucleotide&id=2801887476) |
