# Supplementary material for: Developing molecular classifiers to detect environmental stressors and smolt stages in sockeye salmon, Oncorhynchus nerka
Source: Sci Rep. 2026 Jun 4;16:25584. doi: 10.1038/s41598-026-49226-3 (PMC13478211; doi:10.1038/s41598-026-49226-3)
Supplement: Supplementary file 5 [file 41598_2026_49226_MOESM5_ESM.docx]

Supplementary Table 2. PCR efficiency for each assay per chip tested on Sockeye salmon

| Biomarkers | PCR Efficiency | | | | | | | | | | | | | | | | |
| --- | --- | --- | --- | --- | --- | --- | --- | --- | --- | --- | --- | --- | --- | --- | --- | --- | --- |
|  | Chip1 | Chip2 | Chip3 | Chip4 | Chip5 | Chip6 | Chip7 | Chip8 | Chip9 | Chip10 | Chip11 | Chip12 | Chip13 | Chip14 | Chip15 | Chip16 |  |
| CL_H2EB1_672 | 1.07 | 1.05 | 0.99 | 1.09 | 1.08 | 1.21 | 1.03 | 1.08 | 1.05 | 1.13 | 1.07 | 1.04 | 1.08 | 1.07 | 1.06 | 0.95 |  |
| CL_HLA21.G1_684 | 0.95 | 0.94 | 1.14 | 1.05 | 0.98 | 1.08 | 1.06 | 0.97 | 1.02 | 0.97 | 1.02 | 1.01 | 1.05 | 1.19 | 1.04 | 0.92 |  |
| CL_PSMB8_680 | 0.88 | 1.00 | 1.18 | 0.98 | 1.09 | 1.00 | 0.80 | 0.84 | 0.91 | 0.78 | 1.01 | 1.02 | 0.93 | 0.85 | 0.80 | 0.82 |  |
| GS_HSP90b_268 | 0.81 | 0.92 | 1.14 | 0.88 | 1.08 | 1.12 | 1.11 | 0.98 | 0.97 | 0.92 | 1.00 | 1.00 | 1.15 | 1.28 | 1.01 | 0.82 |  |
| HK_CCDC84_114 | 0.97 | 0.82 | 1.01 | 0.98 | 1.13 | 0.96 | 0.94 | 1.04 | 1.14 | 1.10 | 1.08 | 0.98 | 0.89 | 1.14 | 1.17 | 0.99 |  |
| HK_MRPL40_113 | 0.89 | 1.02 | 0.90 | 1.12 | 1.21 | 1.18 | 1.15 | 0.92 | 0.92 | 1.05 | 1.22 | 1.01 | 1.11 | 1.19 | 1.04 | 0.85 |  |
| HK_S100_10 | 0.98 | 0.98 | 1.01 | 1.05 | 1.21 | 1.16 | 1.24 | 1.01 | 1.26 | 1.14 | 1.21 | 1.00 | 1.12 | 1.53 | 1.07 | 1.02 |  |
| HP_ALDOA1_687 | 0.95 | 1.01 | 1.01 | 1.02 | 1.12 | 1.10 | 0.94 | 1.07 | 1.07 | 0.97 | 1.05 | 1.12 | 1.07 | 1.11 | 0.97 | 0.91 |  |
| HP_ANLN_633 | 0.85 | 0.90 | 1.02 | 1.05 | 1.10 | 1.12 | 1.09 | 1.02 | 0.97 | 1.04 | 1.00 | 0.87 | 0.92 | 1.15 | 0.86 | 0.88 |  |
| HP_ARGR_648 | 0.95 | 0.97 | 1.02 | 1.09 | 1.16 | 1.10 | 1.12 | 0.88 | 0.93 | 1.09 | 0.88 | 1.13 | 0.98 | 1.05 | 0.97 | 0.92 |  |
| HP_AURKB_634 | 0.99 | 0.96 | 0.90 | 1.10 | 1.12 | 1.15 | 1.06 | 0.99 | 1.07 | 0.96 | 1.01 | 0.91 | 1.04 | 1.22 | 0.93 | 0.94 |  |
| HP_CDKN1B_625 | 1.10 | 0.96 | 0.91 | 0.86 | 1.06 | 0.90 | 1.02 | 0.92 | 0.94 | 0.85 | 0.93 | 1.02 | 0.91 | 0.87 | 0.92 | 1.03 |  |
| HP_CIT_636 | 0.87 | 0.98 | 0.97 | 1.05 | 0.95 | 1.10 | 1.05 | 0.97 | 0.89 | 1.02 | 0.97 | 1.08 | 0.99 | 1.03 | 1.01 | 0.83 |  |
| HP_CLSPN_637 | 1.10 | 1.10 | 1.30 | 1.16 | 1.25 | 1.58 | 0.95 | 1.01 | 1.03 | 1.10 | 1.13 | 1.04 | 1.07 | 1.18 | 1.15 | 0.93 |  |
| HP_COX6B_216 | 1.19 | 0.96 | 1.15 | 1.02 | 1.34 | 1.11 | 1.01 | 0.86 | 0.81 | 1.09 | 1.12 | 0.94 | 1.05 | 0.94 | 0.89 | 0.91 |  |
| HP_ECE2_626 | 1.00 | 0.94 | 1.01 | 1.16 | 1.04 | 1.14 | 1.08 | 0.89 | 0.96 | 0.87 | 1.09 | 1.09 | 0.95 | 1.08 | 0.94 | 0.96 |  |
| HP_ENO2_607 | 0.90 | 0.93 | 1.00 | 1.01 | 1.00 | 1.07 | 1.00 | 0.88 | 1.03 | 0.96 | 1.00 | 0.95 | 1.07 | 1.12 | 0.97 | 0.84 |  |
| HP_ERCR_639 | 0.96 | 1.00 | 0.98 | 1.04 | 0.98 | 1.18 | 1.20 | 0.94 | 1.07 | 1.15 | 0.85 | 1.15 | 0.99 | 1.13 | 1.04 | 0.97 |  |
| HP_GP1_608 | 0.93 | 0.90 | 0.97 | 1.04 | 1.08 | 1.10 | 0.99 | 1.10 | 0.99 | 1.01 | 0.94 | 0.92 | 1.06 | 1.06 | 0.94 | 0.94 |  |
| HP_GP4_609 | 0.91 | 0.95 | 1.01 | 1.05 | 1.07 | 1.10 | 1.02 | 0.89 | 1.02 | 0.95 | 1.07 | 1.04 | 1.13 | 1.24 | 0.98 | 0.99 |  |
| HP_H1F1A.7_263 | 1.04 | 0.97 | 1.08 | 1.07 | 1.13 | 1.06 | 1.06 | 0.96 | 1.11 | 1.05 | 1.03 | 1.02 | 1.10 | 1.30 | 1.07 | 1.02 |  |
| HP_H2F2_640 | 0.98 | 0.87 | 0.94 | 0.96 | 0.94 | 0.94 | 1.03 | 0.99 | 1.05 | 1.00 | 0.93 | 0.96 | 0.96 | 1.09 | 0.96 | 0.87 |  |
| HP_HBA_616 | 1.09 | 1.06 | 1.17 | 1.19 | 1.39 | 1.17 | 1.12 | 1.12 | 1.35 | 1.10 | 1.15 | 1.12 | 1.22 | 1.70 | 1.18 | 1.19 |  |
| HP_HIF1A_262 | 1.08 | 1.10 | 1.03 | 0.98 | 1.01 | 1.16 | 1.09 | 0.95 | 0.89 | 1.07 | 0.60 | 0.95 | 0.94 | 1.21 | 0.89 | 1.01 |  |
| HP_HMOX2_612 | 0.91 | 1.09 | 1.11 | 0.98 | 1.17 | 1.12 | 1.13 | 1.03 | 1.14 | 1.10 | 1.20 | 1.05 | 1.06 | 1.10 | 1.12 | 1.05 |  |
| HP_IGFBP1B_284 | 1.04 | 0.95 | 0.93 | 1.03 | 1.03 | 0.99 | 0.98 | 1.01 | 0.99 | 0.94 | 0.94 | 1.01 | 0.98 | 0.99 | 0.98 | 0.95 |  |
| HP_KI1I_641 | 1.02 | 0.92 | 0.95 | 0.88 | 0.98 | 1.01 | 0.99 | 0.93 | 0.95 | 0.95 | 0.94 | 0.82 | 1.12 | 0.95 | 0.98 | 0.84 |  |
| HP_KIF4_635 | 0.98 | 0.85 | 0.98 | 0.94 | 1.16 | 0.84 | 0.85 | 0.96 | 0.96 | 1.00 | 1.05 | 1.00 | 0.77 | 1.38 | 1.07 | 0.75 |  |
| HP_MFHAS1_629 | 1.14 | 0.91 | 1.05 | 1.10 | 1.25 | 1.16 | 1.00 | 0.91 | 1.00 | 1.00 | 1.16 | 0.92 | 0.97 | 1.42 | 1.21 | 0.93 |  |
| HP_NCAPD3_645 | 0.91 | 1.03 | 0.98 | 0.89 | 1.03 | 1.13 | 0.98 | 1.09 | 1.05 | 0.81 | 0.91 | 1.02 | 1.01 | 1.21 | 0.92 | 0.85 |  |
| HP_NDC80_644 | 1.00 | 1.02 | 0.96 | 1.05 | 1.07 | 0.97 | 1.53 | 1.10 | 1.17 | 0.91 | 0.93 | 0.99 | 0.79 | 1.40 | 1.00 | 0.88 |  |
| HP_NGB1_613 | 0.80 | 0.71 | 0.80 | 1.03 | 1.02 | 1.18 | 1.14 | 0.93 | 0.82 | 0.90 | 0.94 | 1.04 | 0.87 | 0.96 | 1.07 | 0.67 |  |
| HP_PAM_630 | 0.93 | 0.96 | 1.04 | 1.06 | 1.12 | 1.02 | 1.06 | 1.03 | 0.96 | 1.01 | 1.00 | 0.99 | 1.03 | 1.14 | 1.08 | 0.99 |  |
| HP_PGK_642 | 0.96 | 0.96 | 0.86 | 1.07 | 1.00 | 0.89 | 0.86 | 0.94 | 0.92 | 0.98 | 0.97 | 0.83 | 1.03 | 1.00 | 0.94 | 0.99 |  |
| HP_PgK3_343 | 1.09 | 1.09 | 1.21 | 1.22 | 1.17 | 1.14 | 1.05 | 1.01 | 1.08 | 1.05 | 1.14 | 1.02 | 1.09 | 1.42 | 1.18 | 1.01 |  |
| HP_RAMP1_631 | 1.16 | 0.86 | 0.90 | 1.03 | 1.01 | 0.86 | 1.00 | 1.00 | 0.80 | 1.01 | 0.85 | 0.90 | 0.96 | 0.85 | 0.99 | 0.93 |  |
| HP_RR2R_650 | 0.94 | 0.94 | 0.92 | 0.97 | 1.04 | 1.05 | 1.00 | 1.06 | 1.09 | 0.98 | 0.91 | 0.97 | 1.03 | 0.86 | 0.92 | 0.89 |  |
| HP_SM4M_651 | 0.76 | 1.04 | 0.99 | 1.02 | 1.16 | 0.98 | 1.14 | 0.96 | 0.85 | 1.19 | 1.02 | 0.95 | 1.01 | 1.10 | 1.04 | 0.92 |  |
| HP_SOX5_632 | 0.93 | 1.00 | 1.00 | 1.07 | 1.07 | 1.06 | 1.06 | 1.04 | 0.92 | 0.88 | 1.06 | 1.05 | 0.98 | 1.22 | 0.91 | 0.79 |  |
| HP_TOSO_652 | 0.91 | 0.83 | 1.17 | 0.93 | 1.17 | 1.16 | 1.13 | 0.99 | 0.88 | 1.06 | 1.02 | 1.00 | 0.97 | 1.08 | 1.00 | 0.98 |  |
| HYP_FAM214A_627 | 0.96 | 1.01 | 1.04 | 0.91 | 1.11 | 1.10 | 1.10 | 1.04 | 1.01 | 0.95 | 1.02 | 0.99 | 1.03 | 1.10 | 1.08 | 0.88 |  |
| IF_IL17D_294 | 1.32 | 1.12 | 1.77 | 1.19 | 1.13 | 0.94 | 1.21 | 1.15 | 1.70 | 1.20 | 1.24 | 1.05 | 1.43 | 1.81 | 1.89 | 1.10 |  |
| IF_MMP13_315 | 1.03 | 0.88 | 1.02 | 1.05 | 1.11 | 0.94 | 0.96 | 0.95 | 1.05 | 1.00 | 0.87 | 1.04 | 0.97 | 1.10 | 1.03 | 0.95 |  |
| IM_ARRDC2_663 | 1.14 | 1.12 | 1.23 | 1.13 | 1.30 | 1.12 | 1.17 | 1.04 | 1.17 | 1.11 | 1.02 | 1.06 | 1.22 | 1.47 | 1.20 | 1.13 |  |
| IM_CLEC4E_666 | 0.90 | 1.03 | 1.15 | 1.02 | 1.13 | 1.13 | 1.83 | 1.05 | 1.13 | 1.11 | 1.05 | 1.86 | 1.14 | 2.08 | 1.11 | 0.92 |  |
| IM_GLUL_670 | 0.90 | 0.94 | 1.01 | 1.06 | 1.03 | 1.02 | 0.98 | 0.89 | 1.07 | 0.98 | 1.00 | 0.93 | 1.06 | 1.14 | 0.97 | 0.90 |  |
| IM_GPX3_628 | 0.97 | 0.85 | 1.03 | 1.03 | 1.14 | 0.96 | 0.97 | 1.07 | 1.04 | 0.94 | 0.95 | 0.84 | 1.12 | 1.21 | 1.00 | 0.83 |  |
| IM_H1F0_671 | 0.95 | 0.88 | 1.03 | 0.95 | 1.48 | 1.11 | 1.05 | 0.88 | 1.03 | 0.96 | 0.84 | 0.93 | 0.99 | 1.09 | 0.96 | 0.79 |  |
| IM_HSP90ab.15_270 | 0.88 | 0.90 | 1.01 | 1.01 | 1.25 | 1.03 | 1.09 | 0.92 | 0.97 | 1.05 | 0.95 | 0.93 | 1.06 | 0.98 | 0.98 | 0.94 |  |
| IM_IQGAP1_675 | 0.86 | 0.94 | 0.99 | 1.09 | 1.06 | 1.08 | 1.13 | 0.90 | 0.97 | 0.96 | 1.06 | 0.90 | 1.00 | 1.18 | 1.07 | 0.81 |  |
| IM_LDH1_306 | 0.86 | 1.03 | 1.05 | 1.03 | 0.70 | 1.19 | 1.02 | 0.87 | 0.83 | 0.83 | 1.00 | 1.01 | 1.32 | 1.17 | 1.03 | 0.95 |  |
| IM_NUPR1_677 | 0.97 | 0.96 | 0.99 | 1.13 | 1.00 | 1.04 | 1.00 | 1.08 | 1.06 | 1.01 | 0.96 | 1.01 | 1.05 | 1.20 | 1.02 | 0.93 |  |
| IM_ODC1_678 | 1.16 | 0.96 | 1.00 | 0.97 | 1.06 | 0.92 | 1.00 | 1.03 | 0.96 | 0.90 | 0.97 | 1.03 | 0.97 | 1.01 | 0.96 | 1.07 |  |
| IM_TAGLN3_681 | 0.84 | 1.14 | 1.08 | 1.04 | 1.15 | 1.23 | 1.23 | 0.94 | 1.17 | 1.12 | 1.02 | 1.04 | 1.07 | 1.39 | 1.13 | 0.90 |  |
| MRS_ATP5G3_181 | 1.02 | 0.91 | 0.94 | 1.03 | 0.98 | 0.91 | 1.10 | 1.00 | 0.94 | 0.94 | 1.03 | 0.99 | 0.99 | 1.06 | 1.00 | 0.89 |  |
| MRS_C7_189 | 1.16 | 0.90 | 0.85 | 0.88 | 0.94 | 0.71 | 1.02 | 1.02 | 0.83 | 0.82 | 0.89 | 1.04 | 0.93 | 0.85 | 0.91 | 0.80 |  |
| MRS_FYB_241 | 1.08 | 0.89 | 0.99 | 0.96 | 1.11 | 0.85 | 1.15 | 1.02 | 0.95 | 0.85 | 0.96 | 0.86 | 1.00 | 1.15 | 1.04 | 1.01 |  |
| MRS_HTATIP_272 | 1.09 | 1.00 | 0.92 | 1.00 | 1.03 | 0.81 | 0.90 | 0.91 | 0.92 | 0.88 | 0.87 | 1.13 | 1.09 | 1.17 | 0.92 | 1.04 |  |
| MRS_IRF1_299 | 0.92 | 1.04 | 1.08 | 1.50 | 1.10 | 1.18 | 1.08 | 0.93 | 0.83 | 1.19 | 0.95 | 0.80 | 0.97 | 1.35 | 1.06 | 1.00 |  |
| MRS_KRT2.8_305 | 1.07 | 0.96 | 1.01 | 1.07 | 1.21 | 1.17 | 0.98 | 1.03 | 1.19 | 0.94 | 0.99 | 1.09 | 1.17 | 1.24 | 0.96 | 0.95 |  |
| MRS_NKAB2_328 | 1.29 | 1.01 | 1.01 | 0.90 | 1.40 | 0.92 | 1.18 | 1.12 | 1.18 | 1.08 | 1.15 | 0.94 | 1.28 | 1.88 | 1.07 | 0.91 |  |
| MRS_PRAS_350 | 0.92 | 0.90 | 0.93 | 1.10 | 1.13 | 1.09 | 1.07 | 0.95 | 0.81 | 1.03 | 0.93 | 1.05 | 1.02 | 1.02 | 0.99 | 0.92 |  |
| MRS_SCG_377 | 1.04 | 0.84 | 0.86 | 0.99 | 0.96 | 1.01 | 0.99 | 1.11 | 0.96 | 1.34 | 0.98 | 0.81 | 0.89 | 0.99 | 0.98 | 1.09 |  |
| OS_CA4_194 | 1.20 | 0.95 | 1.07 | 1.05 | 1.01 | 1.00 | 1.02 | 1.09 | 1.20 | 1.08 | 0.96 | 1.05 | 1.05 | 1.01 | 0.96 | 1.01 |  |
| OS_CCL19_578 | 0.91 | 0.97 | 1.01 | 0.97 | 1.28 | 1.09 | 1.13 | 1.09 | 1.19 | 0.96 | 0.99 | 1.07 | 0.84 | 1.27 | 1.05 | 1.02 |  |
| OS_CCL4_195 | 0.88 | 1.02 | 0.98 | 1.02 | 1.07 | 1.00 | 1.07 | 1.05 | 1.01 | 0.90 | 1.01 | 0.96 | 0.94 | 1.05 | 0.98 | 0.86 |  |
| OS_CEBPB_665 | 0.91 | 0.89 | 0.96 | 0.95 | 1.03 | 0.90 | 0.97 | 0.87 | 0.96 | 0.91 | 0.84 | 1.00 | 1.06 | 0.96 | 0.91 | 0.96 |  |
| OS_CFTR.I_206 | 0.96 | 0.94 | 1.04 | 1.16 | 1.05 | 1.00 | 1.17 | 0.97 | 0.93 | 1.13 | 1.06 | 1.03 | 1.07 | 1.29 | 1.05 | 0.85 |  |
| OS_CLEC4M_211 | 0.95 | 0.94 | 0.98 | 0.93 | 1.04 | 0.93 | 0.97 | 0.92 | 0.99 | 0.99 | 0.96 | 0.90 | 1.05 | 1.07 | 0.96 | 0.83 |  |
| OS_EEF2_580 | 0.91 | 0.97 | 1.02 | 0.99 | 1.05 | 1.06 | 1.02 | 0.98 | 1.07 | 1.06 | 0.98 | 0.98 | 1.11 | 1.18 | 1.03 | 0.84 |  |
| OS_FKBP5_584 | 1.00 | 0.90 | 0.90 | 1.04 | 0.88 | 0.82 | 0.95 | 0.98 | 0.88 | 0.82 | 0.97 | 0.84 | 0.99 | 0.89 | 0.97 | 0.87 |  |
| OS_HBA_254 | 1.10 | 0.98 | 1.11 | 1.06 | 1.19 | 0.95 | 1.23 | 1.14 | 1.09 | 1.00 | 1.15 | 1.11 | 1.03 | 1.40 | 1.06 | 1.14 |  |
| OS_NAKATPASE1a_330 | 1.11 | 1.07 | 1.09 | 1.13 | 1.21 | 1.13 | 1.08 | 1.06 | 1.24 | 1.10 | 1.19 | 1.17 | 1.00 | 1.20 | 1.04 | 1.12 |  |
| OS_NAKATPASE1b_333 | 1.03 | 1.02 | 1.10 | 1.13 | 1.23 | 1.11 | 1.12 | 1.10 | 1.47 | 1.11 | 1.14 | 1.05 | 1.06 | 1.46 | 1.06 | 1.04 |  |
| OS_NAMPT_593 | 1.20 | 1.16 | 1.14 | 1.12 | 1.23 | 1.08 | 1.39 | 1.04 | 1.17 | 1.05 | 1.03 | 1.16 | 1.15 | 1.55 | 1.46 | 1.31 |  |
| OS_NDUFB2_322 | 1.06 | 0.94 | 0.98 | 1.05 | 1.02 | 0.94 | 0.98 | 1.04 | 1.02 | 0.95 | 0.97 | 1.01 | 0.98 | 1.03 | 0.99 | 1.05 |  |
| OS_RGS21_597 | 1.11 | 1.15 | 1.50 | 1.10 | 1.17 | 1.42 | 1.07 | 1.09 | 1.11 | 1.09 | 1.20 | 1.07 | 1.23 | 1.76 | 0.90 | 1.39 |  |
| OS_RHAG_599 | 0.98 | 0.92 | 0.99 | 1.09 | 1.16 | 0.95 | 1.07 | 0.96 | 1.03 | 0.95 | 0.94 | 1.11 | 1.09 | 1.15 | 1.10 | 1.02 |  |
| TM_FKBP10.2_582 | 0.93 | 0.93 | 1.10 | 1.05 | 1.20 | 1.20 | 1.08 | 0.93 | 1.14 | 1.11 | 0.99 | 1.09 | 1.02 | 1.09 | 0.91 | 0.90 |  |
| TM_FKBP10.4_583 | 0.97 | 0.91 | 0.93 | 1.11 | 1.00 | 1.19 | 1.06 | 1.00 | 0.96 | 1.01 | 1.02 | 1.06 | 1.09 | 1.19 | 0.99 | 0.82 |  |
| TM_HSP70_267 | 0.90 | 0.91 | 1.04 | 0.93 | 1.16 | 1.04 | 1.21 | 0.89 | 1.15 | 1.08 | 1.03 | 0.94 | 1.08 | 1.27 | 1.06 | 0.92 |  |
| TM_HSP90a.15_269 | 0.88 | 0.85 | 0.99 | 1.18 | 0.99 | 1.07 | 1.12 | 1.10 | 1.03 | 0.87 | 1.12 | 0.98 | 1.18 | 1.11 | 1.05 | 0.76 |  |
| TM_HSP90a.6_271 | 0.97 | 0.97 | 0.92 | 1.02 | 1.16 | 0.97 | 1.09 | 1.00 | 0.88 | 1.08 | 0.94 | 1.11 | 0.94 | 1.18 | 1.04 | 0.82 |  |
| TM_MAP3K14_308 | 0.99 | 0.91 | 1.00 | 1.04 | 1.27 | 1.15 | 1.13 | 1.09 | 1.04 | 1.03 | 1.12 | 0.98 | 1.01 | 0.99 | 0.95 | 0.87 |  |
| TM_SERPIN20_379 | 0.95 | 0.90 | 0.89 | 1.05 | 1.07 | 1.11 | 1.10 | 0.96 | 0.91 | 0.96 | 0.96 | 0.92 | 0.96 | 1.00 | 0.97 | 0.87 |  |
| TM_SERPIN9_380 | 0.94 | 1.00 | 0.94 | 0.90 | 1.03 | 1.04 | 0.99 | 0.98 | 0.88 | 0.93 | 0.95 | 0.91 | 1.07 | 1.03 | 0.91 | 0.85 |  |
| VDD_GAL3_72 | 1.08 | 1.10 | 1.02 | 1.24 | 1.17 | 1.21 | 1.14 | 1.05 | 1.12 | 1.16 | 0.96 | 1.04 | 1.24 | 1.51 | 1.11 | 1.11 |  |
| VDD_HERC6_77 | 1.49 | 1.04 | 1.05 | 1.18 | 1.28 | 0.95 | 0.88 | 1.01 | 1.15 | 1.13 | 1.09 | 1.18 | 1.35 | 1.74 | 1.16 | 1.10 |  |
| VDD_IFI44A_81 | 0.94 | 0.76 | 0.97 | 0.91 | 1.00 | 0.85 | 1.00 | 0.85 | 1.09 | 0.98 | 1.16 | 0.92 | 1.06 | 1.30 | 0.92 | 0.95 |  |
| VDD_IFIT5.2_83 | 1.00 | 0.88 | 0.97 | 1.01 | 1.07 | 0.92 | 1.12 | 0.88 | 1.08 | 0.99 | 0.98 | 1.06 | 1.09 | 1.14 | 1.00 | 0.90 |  |
| VDD_MX_86 | 0.95 | 0.92 | 0.92 | 0.94 | 1.07 | 1.06 | 1.06 | 1.05 | 0.94 | 0.89 | 0.88 | 0.97 | 0.95 | 1.22 | 1.03 | 0.81 |  |
| VDD_NFX_87 | 1.01 | 1.02 | 1.03 | 0.98 | 1.03 | 0.99 | 0.96 | 0.95 | 0.88 | 1.03 | 0.94 | 1.05 | 0.99 | 1.13 | 0.88 | 0.96 |  |
| VDD_RSAD_367 | 0.94 | 1.10 | 1.12 | 0.94 | 1.11 | 1.13 | 1.12 | 1.15 | 1.03 | 1.20 | 1.06 | 1.02 | 1.13 | 1.24 | 0.97 | 0.88 |  |
| VDD_TRIM1_409 | 1.00 | 1.01 | 1.03 | 1.05 | 1.05 | 1.15 | 1.10 | 1.10 | 1.14 | 1.11 | 0.98 | 1.07 | 1.02 | 1.16 | 1.05 | 1.02 |  |
