# Supplementary material for: Developing molecular classifiers to detect environmental stressors and smolt stages in sockeye salmon, Oncorhynchus nerka
Source: Sci Rep. 2026 Jun 4;16:25584. doi: 10.1038/s41598-026-49226-3 (PMC13478211; doi:10.1038/s41598-026-49226-3)
Supplement: Supplementary file 6 [file 41598_2026_49226_MOESM6_ESM.docx]

Supplementary Table 3. The preliminary analysis to select the ultimate biomarkers for thermal stress classification analysis. ANOVA/Tukey tests were conducted preliminary on the data to find the significant biomarkers. Then, the initial Random Forest-based model classification was applied on all the significant biomarkers to rank and select the ultimate biomarkers for final RF classification. P values are FDR-adjusted.

| Gene name | ANOVA/Tukey | Passed for initial RF | Biomarker ranks after initial RF | Biomarker selected for final RF |
| --- | --- | --- | --- | --- |
| TM_HSP70_267 | P = 1.4 × 10^-6^ 10_14°C; P = 0.117  10_18°C; P < 0.001 14_18°C; P < 0.001 | √ | Rank 10 | **-** |
| TM_HSP90AA_269 | P = 1.92 × 10^-7^ 10_14°C; P < 0.001  10_18°C; P < 0.001 14_18°C; P < 0.001 | √ | Rank 7 | √ |
| TM_HSP90a_271 | P = 1.92 × 10^-7^ 10_14°C; P < 0.001  10_18°C; P < 0.001 14_18°C; P < 0.001 | √ | Rank 3 | √ |
| TM_MAP3K14_308 | P = 0.0001 10_14°C; P < 0.01  10_18°C; P < 0.001 14_18°C; P < 0.001 | √ | Rank 9 | √ |
| TM_FKBP10_2_582 | P = 0.0441 10_14°C; P < 0.001  10_18°C; P < 0.001 14_18°C; P < 0.001 | √ | Rank 6 | √ |
| TM_FKBP10_4_583 | P = 0.0441 10_14°C; P < 0.001  10_18°C; P < 0.001 14_18°C; P < 0.001 | √ | Rank 8 | √ |
| TM_SERPIN_20_379 | P = 1.92 × 10^-7^ 10_14°C; P < 0.001  10_18°C; P < 0.001 14_18°C; P < 0.001 | √ | Rank 1 | √ |
| TM_SERPIN_9_380 | P = 1.92 × 10^-7^ 10_14°C; P < 0.001  10_18°C; P < 0.001 14_18°C; P < 0.001 | √ | Rank 2 | √ |
| OS_EEF2_580 | P = 4.27× 10^-5^ 10_14°C; P < 0.001  10_18°C; P < 0.001 14_18°C; P < 0.001 | √ | Rank 4 | √ |
| IM_HSP90ab_270* | P = 0.0002 10_14°C; P < 0.001  10_18°C; P < 0.001 14_18°C; P < 0.001 | √ | Rank 11 | **-** |
| GS_HSP90b_268* | P = 1.92 × 10^-7^ 10_14°C; P < 0.001  10_18°C; P < 0.001 14_18°C; P < 0.001 | √ | Rank 5 | √ |
| Sensitivity scores for 1/3 testing data (threshold 0.5) | | | 1-10°C = 100.0%  2-14°C = 90.0%  3-18°C = 100.0% | 1-10°C = 100.0%  2-14°C = 90.0%  3-18°C = 100.0% |

* These biomarkers from other panels were selected for initial RF analysis given their function and significant P value.
