# Supplementary material for: Developing molecular classifiers to detect environmental stressors and smolt stages in sockeye salmon, Oncorhynchus nerka
Source: Sci Rep. 2026 Jun 4;16:25584. doi: 10.1038/s41598-026-49226-3 (PMC13478211; doi:10.1038/s41598-026-49226-3)
Supplement: Supplementary file 7 [file 41598_2026_49226_MOESM7_ESM.docx]

Supplementary Table 4. The preliminary analysis to select the ultimate biomarkers for hypoxic stress classification analysis. T-test (normality passed) and Mann-Whitney non-parametric t-test (normality failed) and Receiver Operating Characteristic (ROC) analyses were conducted preliminary on the 2/3 training data to find the significant biomarkers. Then, the initial Random Forest-based model classification was applied on all the significant biomarkers using the training data to rank and select the ultimate biomarkers for final RF classification. Sensitivity scores were calculated for 1/3 of the testing data to demonstrate the performance of the Random Forest (RF) model in both the initial and final analyses. P values are FDR-adjusted.

| Gene name | T-test | AUC score | Passed for initial RF | Biomarker ranks after initial RF | Biomarker selected for final RF |
| --- | --- | --- | --- | --- | --- |
| HP_HIF1A_262 | P = 0 | 0.8833 | √ | Rank 1 | √ |
| HP_AURKB_634 | P = 0 | 0.8125 | √ | Rank 3 | √ |
| HP_RAMP1_631 | P = 0 | 0.799 | √ | Rank 2 | √ |
| HP_KI1I_641 | P = 0 | 0.7784 | √ | Rank 4 | √ |
| HP_NCAPD3_645 | P = 8.08×10^-15^ | 0.7696 | √ | Rank 8 | √ |
| HP_ARGR_648 | P = 2.46×10^-13^ | 0.7555 | √ | Rank 11 | - |
| HP_RR2R_650 | P = 6.32×10^-13^ | 0.7555 | √ | Rank 6 | √ |
| HP_CIT_636 | P = 1.06×10^-12^ | 0.7479 | √ | Rank 10 | - |
| HP_KIF4_635 | P = 1.66×10^-11^ | 0.735 | √ | Rank 13 | **-** |
| HP_CLSPN_637 | P = 2.11×10^-11^ | 0.7334 | √ | Rank 14 | - |
| HP_NDC80_644 | P = 2.23×10^-11^ | 0.7327 | √ | Rank 19 | **-** |
| HP_ERCR_639 | P = 1.58×10^-10^ | 0.723 | √ | Rank 15 | - |
| HP_ECE2_626 | P = 1.89×10^-10^ | 0.7217 | √ | Rank 5 | √ |
| HP_TOSO_652 | P = 1.95×10^-9^ | 0.7096 | √ | Rank 21 | **-** |
| HP_PAM_630 | P = 2.17×10^-9^ | 0.7087 | √ | Rank 9 | - |
| HP_PGK_642 | P = 2.94×10^-8^ | 0.6944 | √ | Rank 26 | - |
| HP_ENO2_607 | P = 3.34×10^-8^ | 0.6934 | √ | Rank 7 | √ |
| HP_ANLN_633 | P = 4.15×10^-8^ | 0.6918 | √ | Rank 16 | **-** |
| OS_RGS21_597 * | P = 5.79×10^-6^ | 0.6616 | √ | Rank 17 | **-** |
| VDD_IFIT5-2_83 * | P = 6.74×10^-6^ | 0.6602 | √ | Rank 20 | **-** |
| HP_H2F2_640 | P = 2.25×10^-5^ | 0.6517 | √ | Rank 18 | **-** |
| IF_MMP13_315 * | P = 0.0002 | 0.6348 | √ | Rank 27 | **-** |
| HP_CDKN1B_625 | P = 0.0072 | 0.6038 | √ | Rank 24 | **-** |
| OS_CLEC4M_211 * | P = 0.0075 ** | 0.603 | √ | Rank 23 | **-** |
| HP_SM4M_651 | P = 0.0117 | 0.598 | √ | Rank 22 | **-** |
| HP_MFHAS1_629 | P = 0.0309 | 0.5872 | √ | Rank 25 | **-** |
| OS_CA4_194 * | P = 0.0426 | 0.583 | √ | Rank 12 | **-** |
| CL_HLA21-G1_684 * | P = 0.0168 ** | 0.5796 | √ | Rank 28 | **-** |
| Sensitivity scores for 1/3 testing data (threshold 0.5) | | | | 1-Normoxia = 87.7%  2-Hypoxia = 81.9% | 1-Normoxia = 87.7%  2-Hypoxia = 83.3% |

* biomarkers from other panels were selected for initial RF analysis given their function and significant P value.

** Normality failed; Data were tested using the Mann-Whitney non-parametric t-test.
