# Supplementary material for: Developing molecular classifiers to detect environmental stressors and smolt stages in sockeye salmon, Oncorhynchus nerka
Source: Sci Rep. 2026 Jun 4;16:25584. doi: 10.1038/s41598-026-49226-3 (PMC13478211; doi:10.1038/s41598-026-49226-3)
Supplement: Supplementary file 8 [file 41598_2026_49226_MOESM8_ESM.docx]

Supplementary Table 4. The preliminary analysis to select the ultimate biomarkers for salinity acclimation classification analysis. T-test (normality passed) and Mann-Whitney non-parametric t-test (normality failed) and Receiver Operating Characteristic (ROC) analyses were conducted preliminary on the 2/3 training data to find the significant biomarkers. Then, the initial Random Forest-based model classification was applied on all the significant biomarkers using the training data to rank and select the ultimate biomarkers for final RF classification. Sensitivity scores were calculated for 1/3 of the testing data to demonstrate the performance of the Random Forest (RF) model in both the initial and final analyses. P values are FDR-adjusted.

| Gene name | T-test | AUC score | Passed for initial RF | Biomarker ranks after initial RF | | Biomarker selected for final RF | |  |
| --- | --- | --- | --- | --- | --- | --- | --- | --- |
| OS_NAKATPASE1b_333 | P = 0 | 0.9359 | √ | Rank 2 | | √ | |  |
| OS_NAKATPASE1a_330 | P = 0 ** | 0.9049 | √ | Rank 1 | | √ | |  |
| HP_KI1I_641 | P = 4.09 × 10^-9^ ** | 0.7663 | √ | Rank 8 | |  | |  |
| OS_CCL4_195 | P = 6.15 × 10^-9^ ** | 0.7619 | √ | Rank 4 | | √ | |  |
| HP_CIT_636 | P = 2.72 × 10^-8^ ** | 0.7497 | √ | Rank 9 | | - | |  |
| MRS_SCG_377 | P = 2.72 × 10^-8^ | 0.7495 | √ | Rank 6 | | - | |  |
| OS_CCL19_578 | P = 6.88 × 10^-8^ ** | 0.7421 | √ | Rank 5 | | √ | |  |
| IF_MMP13_315 | P = 1.4 × 10^-7^ | 0.7362 | √ | Rank 7 | | - | |  |
| VDD_RSAD_367 | P = 1.53 × 10^-7^ ** | 0.7347 | √ | Rank 10 | | - | |  |
| OS_CFTR1_206 | P = 1.82 × 10^-7^** | 0.7327 | √ | Rank 3 | | √ | |  |
| HP_NCAPD3_645 | P = 5.33 × 10^-7^** | 0.7242 | √ | Rank 28 | | - | |  |
| HP_H2F2_640 | P = 6.76 × 10^-7^** | 0.7218 | √ | Rank 11 | | - | |  |
| HP_ERCR_639 | P = 7.17 × 10^-7^** | 0.7207 | √ | Rank 12 | | - | |  |
| HP_NDC80_644 | P = 8.56 × 10^-7^** | 0.7188 | √ | Rank 14 | | - | |  |
| HP_RR2R_650 | P = 1.27 × 10^-6^** | 0.7153 | √ | Rank 32 | | - | |  |
| HP_CLSPN_637 | P = 2.41 × 10^-6^ | 0.7098 | √ | Rank 29 | | - | |  |
| HP_AURKB_634 | P = 2.59 × 10^-6^** | 0.7087 | √ | Rank 23 | | - | |  |
| IM_ARRDC2_663 | P = 2.34 × 10^-5^ | 0.69 | √ | Rank 20 | | - | |  |
| VDD_MX_86 | P = 2.95 × 10^-5^** | 0.6872 | √ | Rank 19 | | - | |  |
| HP_ANLN_633 | P = 2.95 × 10^-5^ | 0.6871 | √ | Rank 26 | | - | |  |
| MRS_KRT2_8_305 | P = 0.0002 ** | 0.6694 | √ | Rank 18 | | - | |  |
| HP_TOSO_652 | P = 0.0002 ** | 0.6678 | √ | Rank 25 | | - | |  |
| HP_ARGR_648 | P = 0.0003 ** | 0.6657 | √ | Rank 27 | | - | |  |
| MRS_IRF1_299 | P = 0.0003 ** | 0.6646 | √ | Rank 17 | | - | |  |
| HP_SOX5_632 | P = 0.0003 | 0.6638 | √ | Rank 15 | | - | |  |
| HP_PGK_642 | P = 0.0006 ** | 0.6565 | √ | Rank 35 | | - | |  |
| IM_ODC1_678 | P = 0.0007 ** | 0.6533 | √ | Rank 21 | | - | |  |
| VDD_IFIT5-2_83 | P = 0.0009 ** | 0.6505 | √ | Rank 33 | | - | |  |
| HP_CDKN1B_625 | P = 0.0011 ** | 0.6479 | √ | Rank 16 | | - | |  |
| VDD_HERC6_77 | P = 0.0012 ** | 0.6471 | √ | Rank 22 | | - | |  |
| MRS_HTATIP_272 | P = 0.0050 ** | 0.6302 | √ | Rank 30 | | - | |  |
| HP_NGB1_613 | P = 0.0104 | 0.6205 | √ | Rank 34 | | - | |  |
| IM_NUPR1_677 | P = 0.0113 | 0.6191 | √ | Rank 31 | | - | |  |
| MRS_NKAB1_328 | P = 0.0235 | 0.6081 | √ | Rank 24 | | - | |  |
| HP_ECE2_626 | P = 0.0235 ** | 0.6079 | √ | Rank 36 | | - | |  |
| HP_H1F1A-7_263 | P = 0.0429 ** | 0.6079 | √ | Rank 13 | | - | |  |
| Sensitivity scores for 1/3 testing data (threshold 0.5) | | | | | 1-FW = 100.0%  2-SW = 97.2% | | 1-FW = 100.0%  2-SW = 100.0% | |

** Normality failed; Data were tested using the Mann-Whitney non-parametric t-test.
