# Supplementary material for: Developing molecular classifiers to detect environmental stressors and smolt stages in sockeye salmon, Oncorhynchus nerka
Source: Sci Rep. 2026 Jun 4;16:25584. doi: 10.1038/s41598-026-49226-3 (PMC13478211; doi:10.1038/s41598-026-49226-3)
Supplement: Supplementary file 9 [file 41598_2026_49226_MOESM9_ESM.docx]

Supplementary Table 5. The preliminary analysis to select the ultimate biomarkers for smolt stage classification analysis. ANOVA test were conducted preliminary on the data to find the significant biomarkers. Then, the initial Random Forest-based model classification was applied on all the significant biomarkers to rank and select the ultimate biomarkers for final RF classification. P values are FDR-adjusted.

| Gene name | ANOVA | Passed for initial RF | Biomarker ranks after initial RF | Biomarker selected for final RF |
| --- | --- | --- | --- | --- |
| OS_CCL4_195 | P < 0.001 | √ | Rank 28 | **-** |
| MRS_ATP5G3_181 | P < 0.001 | √ | Rank 8 | √ |
| HP_RR2R_650 | P < 0.001 | √ | Rank 31 | **-** |
| IM_CLEC4E_666 | P < 0.001 | √ | Rank 6 | √ |
| HP_HBA_616 | P < 0.001 | √ | Rank 9 | √ |
| OS_NDUFB2_322 | P < 0.001 | √ | Rank 12 | **-** |
| MRS_NKAB2_328 | P < 0.001 | √ | Rank 3 | √ |
| VDD_RSAD_367 | P < 0.001 | √ | Rank 5 | √ |
| MRS_SCG_377 | P < 0.001 | √ | Rank 32 | **-** |
| HP_ARGR_648 | P < 0.001 | √ | Rank 19 | **-** |
| HP_H2F2_640 | P < 0.001 | √ | Rank 15 | **-** |
| OS_HBA_254 | P < 0.001 | √ | Rank 14 | **-** |
| OS_CA4_194 | P < 0.001 | √ | Rank 1 | √ |
| HP_NGB1_613 | P < 0.001 | √ | Rank 49 | **-** |
| HP_ERCR_639 | P < 0.001 | √ | Rank 7 | √ |
| IF_MMP13_315 | P < 0.001 | √ | Rank 4 | √ |
| HP_AURKB_634 | P < 0.001 | √ | Rank 39 | **-** |
| IM_TAGLN3_681 | P < 0.001 | √ | Rank 37 | **-** |
| HP_NCAPD3_645 | P < 0.001 | √ | Rank 56 | **-** |
| HP_CIT_636 | P < 0.001 | √ | Rank 53 | **-** |
| OS_CCL19_578 | P < 0.001 | √ | Rank 16 | **-** |
| HP_KI1I_641 | P < 0.001 | √ | Rank 46 | **-** |
| OS_RGS21_597 | P < 0.001 | √ | Rank 11 | **-** |
| IM_LDH1_306 | P < 0.001 | √ | Rank 34 | **-** |
| HP_NDC80_644 | P < 0.001 | √ | Rank 33 | **-** |
| OS_CLEC4M_211 | P < 0.001 | √ | Rank 55 | **-** |
| VDD_MX_86 | P < 0.001 | √ | Rank 47 | **-** |
| TM_HSP90a-15_269 | P < 0.001 | √ | Rank 40 | **-** |
| HP_IGFBP1B_284 | P < 0.001 | √ | Rank 38 | **-** |
| TM_HSP90a-6_271 | P < 0.001 | √ | Rank 36 | **-** |
| HP_HMOX2_612 | P < 0.001 | √ | Rank 22 | **-** |
| HK_MRPL40_113 | P < 0.001 | √ | Rank 10 | √ |
| HP_TOSO_652 | P < 0.001 | √ | Rank 20 | **-** |
| GS_HSP90b_268 | P < 0.001 | √ | Rank 43 | **-** |
| HP_ANLN_633 | P < 0.001 | √ | Rank 48 | **-** |
| IM_ODC1_678 | P < 0.001 | √ | Rank 26 | **-** |
| TM_FKBP10-2_582 | P < 0.001 | √ | Rank 17 | **-** |
| OS_RHAG_599 | P < 0.001 | √ | Rank 35 | **-** |
| MRS_C7_189 | P < 0.001 | √ | Rank 29 | **-** |
| IM_GPX3_628 | P < 0.01 | √ | Rank 25 | **-** |
| HP_SOX5_632 | P < 0.01 | √ | Rank 42 | **-** |
| HP_FAM214A_627 | P < 0.01 | √ | Rank 57 | **-** |
| VDD_IFIT5-2_83 | P < 0.01 | √ | Rank 2 | √ |
| TM_SERPIN20_379 | P < 0.01 | √ | Rank 54 | **-** |
| HP_PAM_630 | P < 0.01 | √ | Rank 27 | **-** |
| HP_PgK3_343 | P < 0.01 | √ | Rank 30 | **-** |
| IF_IL17D_294 | P < 0.01 | √ | Rank 24 | **-** |
| TM_MAP3K14_308 | P < 0.01 | √ | Rank 23 | **-** |
| TM_SERPIN9_380 | P < 0.01 | √ | Rank 41 | **-** |
| HP_MFHAS1_629 | P < 0.01 | √ | Rank 52 | **-** |
| TM_HSP70_267 | P < 0.01 | √ | Rank 44 | **-** |
| VDD_IFI44A_81 | P < 0.01 | √ | Rank 21 | **-** |
| TM_FKBP10-4_583 | P < 0.05 | √ | Rank 45 | **-** |
| HP_PGK_642 | P < 0.05 | √ | Rank 51 | **-** |
| MRS_PRAS_350 | P < 0.05 | √ | Rank 50 | **-** |
| OS_EEF2_580 | P < 0.05 | √ | Rank 18 | **-** |
| OS_NAKATPASE1b_333 | P < 0.05 | √ | Rank 13 | √ |
| Sensitivity scores for 1/3 testing data (threshold 0.5) | | | Pre-smolt = 45.5%  Smolt = 91.7%  De-smolt = 88.5% | Pre-smolt = 72.7%  Smolt = 88.1%  De-smolt = 88.5% |

* These biomarkers from other panels were selected for initial RF analysis given their function and significant P value.
